# High-Resolution Disease Phenotyping Reveals Distinct Resistance Mechanisms of Tomato Crop Wild Relatives against *Sclerotinia sclerotiorum*

**DOI:** 10.34133/plantphenomics.0214

**Published:** 2024-08-05

**Authors:** Severin Einspanier, Christopher Tominello-Ramirez, Mario Hasler, Adelin Barbacci, Sylvain Raffaele, Remco Stam

**Affiliations:** ^1^ Department of Phytopathology and Crop Protection, Institute of Phytopathology, Faculty of Agricultural and Nutritional Sciences, Christian-Albrechts-University, 24118 Kiel, Germany.; ^2^ Lehrfach Variationsstatistik, Faculty of Agricultural and Nutritional Sciences, Christian-Albrechts-University, Kiel, 24118 Kiel, Germany.; ^3^ Laboratoire des Interactions Plantes Microorganismes Environnement (LIPME), INRAE, CNRS, Castanet Tolosan Cedex, France.

## Abstract

Besides the well-understood qualitative disease resistance, plants possess a more complex quantitative form of resistance: quantitative disease resistance (QDR). QDR is commonly defined as a partial but more durable form of resistance and, therefore, might display a valuable target for resistance breeding. The characterization of QDR phenotypes, especially of wild crop relatives, displays a bottleneck in deciphering QDR’s genomic and regulatory background. Moreover, the relationship between QDR parameters, such as infection frequency, lag-phase duration, and lesion growth rate, remains elusive. High hurdles for applying modern phenotyping technology, such as the low availability of phenotyping facilities or complex data analysis, further dampen progress in understanding QDR. Here, we applied a low-cost (<1.000 €) phenotyping system to measure lesion growth dynamics of wild tomato species (e.g., *Solanum pennellii* or *Solanum pimpinellifolium*). We provide insight into QDR diversity of wild populations and derive specific QDR mechanisms and their cross-talk*.* We show how temporally continuous observations are required to dissect end-point severity into functional resistance mechanisms. The results of our study show how QDR can be maintained by facilitating different defense mechanisms during host–parasite interaction and that the capacity of the QDR toolbox highly depends on the host’s genetic context. We anticipate that the present findings display a valuable resource for more targeted functional characterization of the processes involved in QDR. Moreover, we show how modest phenotyping technology can be leveraged to help answer highly relevant biological questions.

## Introduction

### Quantitative disease resistance in plants

Plant resistance is commonly divided into 2 concepts with fundamental differences: qualitative and quantitative resistance [1,2]. While qualitative disease resistance provides a highly effective race-specific resistance, quantitative disease resistance (QDR) is a broad-range yet incomplete resistance [2,3]. Qualitative resistance is driven by major race-specific resistance genes (R-genes). They often lead to complete and easily observable resistance and were the dominant research focus for disease resistance breeding programs. However, reports of R-genes losing their efficacy against pathogens have increased recently, and major resistance genes have not been identified for many so-called necrotrophic plant pathogens, like *Botrytis cinerea* or *Sclerotinia sclerotiorum* [3–7]*.* Commonly, degrees of QDR cannot be divided into discrete classes. Quantitative resistance phenotypes are continuously distributed and can only be explained by highly integrated, polygenic regulatory mechanisms [8]. Moreover, QDR can manifest itself in several ways, ranging from differences in infection frequency (IF) on the leaf or delayed onset of infection to stalled lesion growth. Numerous studies documented wide distributions of QDR phenotypes against necrotrophic pathogens in both natural and domesticated plant populations, yet the relations of different QDR phenotypes have not yet been studied in detail [1–3,8–12]. Recent reports summarized the diversity in functional QDR, arguing that QDR might be influenced by many independent components such as regulation as a pleiotropic side effect, weak R-genes, involvement in defense signal transduction, or *cis*/*trans*-regulatory mechanisms [1,2]. Indeed, many QTLs that influence some degree of QDR have been identified [8,13,14]. Linkage of such QTLs or the underlying loci to exact resistance features, like the lag-phase duration, will be one of the future challenges that would allow understanding and utilizing QDR in pathogen resistance breeding.

### Phenotyping technology and approaches to quantify QDR

The functional characterization of QDR highly depends on precisely measured phenotypes [2,15]. However, the experimental design required to assess QDR phenotypes over entire plant or pathogen populations quickly exceeds the limits of traditional, manual scoring methods and calls for more sophisticated phenotyping technology. The increasing availability of sensor technology (e.g., RGB, multi- or hyperspectral sensors) and analytical methods (e.g., deep-learning or artificial intelligence algorithms) recently have strengthened the attention to plant phenotyping [16]. Many studies have shown how imaging technology can be used not only to determine plant phenotypes like plant height, nutritional status, or water-use efficiency but also to assist breeder’s decisions [14,17,18]. Moreover, several reviews recently summarized the potential of modern sensor technology and related software in quantifying phenotypes of host–parasite interactions on multiple levels [19–24]. Even advanced applications, like in-field phenotyping or assessing complex features in non-standardized conditions, are possible due to deep-learning models like “PLPNet” or “ResNet-9” [25–27]. However, large phenotyping platforms also have limitations. High-end systems often collect a multitude of 3D scanning images or images in multiple spectral wavelengths. Analysis of these data is computationally intensive and often requires very specific knowledge. Thus, such technologies might overwhelm (non-data-science) researchers with high amounts of complex datasets as substantial skills are required to derive easy-to-interpret insights relevant to answering biological research questions [28]. A second challenge lies in adapting an established phenotyping system for various pathosystems, i.e., different crops or pathogens [22,29]. Lastly, most high-end phenotyping systems have very high investment and running costs and thus are less available. Combined with the aforementioned low flexibility, this further limits their use and application in the broad spectrum of plant pathology, where quick and easy screening of QDR in a large panel of plants is one of the main objectives. Recent developments, however, enable researchers to use the generally available consumer-level technology and build low-cost phenotyping platforms like the “Navautron” [30]. In this study, we show the usefulness of such systems in unraveling QDR dynamics in crop wild relatives.

### Wild tomato populations as a reservoir of potential QDR loci against major pathogens

The domestic tomato (*Solanum lycopersicum*) is a major food crop of global importance [31]. However, plant pathogens, including the necrotroph *S. sclerotiorum* or species from the genus *Alternaria*, commonly threaten tomato production worldwide [32–35]. Host resistance and fungicides are the standard tools to protect tomatoes against these pathogens. However, strong selection pressure caused by R-genes or fungicides and higher-than-expected pathogen diversity in the field result in losing fungicide efficacy or plant resistance against such species [36–40]. Therefore, highly diverse wild populations are an invaluable source of desirable alleles in breeding, as crosses between wild and domestic can lead to increased performance and stress tolerance [41]. Integrating phenotyping with screening of genetically highly diverse wild resources will help characterize novel alleles for QDR breeding [42].

Wild tomato species originated from several radiation events and can generally be classified into 4 groups within the so-called section Lycopersicon, containing a total of 15 species and 2 species in the section Lycopersicoides [43]. All species have adapted to specific habitats ranging from the edge of the Atacama desert to the Andes, where they withstand diverse (a)biotic stresses. Evolutionary analyses show that different species and populations have evolved drought or salt stress tolerance, as well as adaptation to cold stress [44–48]. Previous studies have also shown substantial variation in susceptibility and resistance of wild *Solanum* spp. against various pathogens but often relied on manual or single time-point disease assessments, thus lacking the temporal resolution and statistical power to describe QDR mechanisms confidently [38,49,50]. In light of the variation of QDR already shown, wild tomato species are perfectly suited for quantification of QDR mechanisms as proof of principle. Moreover, defining whether specific QDR mechanisms play major roles in resistance will generate much-needed insights into the biology of QDR to help design future durable resistance breeding projects against major pathogens.

*S. sclerotiorum* is a necrotrophic pathogen that can infect hundreds of host species, including important crops such as rapeseed and tomato [30,51,52]. On vegetables, including tomatoes, infection with *S. sclerotiorum* can cause tremendous yield loss due to collapsing stems or damaged fruits [53,54]. Infection in the field can happen through air-dispersed ascospores or via myceliogenic germination of its overwintering structures in the soil, the so-called sclerotia [32,52]. In experimental conditions, mycelial inoculation procedures are commonly used, as the preparation of ascospores can display a major challenge [55–59]. No complete form of resistance against the generalist *S. sclerotiorum* has been characterized; therefore, resistance breeding relies on QDR as the source of new alleles [32,52,55,57,60].

In the present work, we build on a low-budget image-based phenotyping system [30] to derive high-resolution time-resolved disease phenotypes and dissect them into 3 distinct QDR mechanisms. We show the potential of this system by characterizing the natural diversity of QDR phenotypes of wild *Solanum* species and, therefore, provide insights into the mechanisms underlying QDR against the generalist pathogen *S. sclerotiorum*. We use this system as a model to address whether QDR is always represented by a similar mechanism, i.e., IF or lag-phase duration, and show that the orchestration of different QDR mechanisms affects the overall QDR on a genotype-specific basis. Accordingly, we argue that the different host species have evolved specific mechanisms to maintain a defined degree of QDR.

## Materials and Methods

### Experimental design

**Fig.1.**
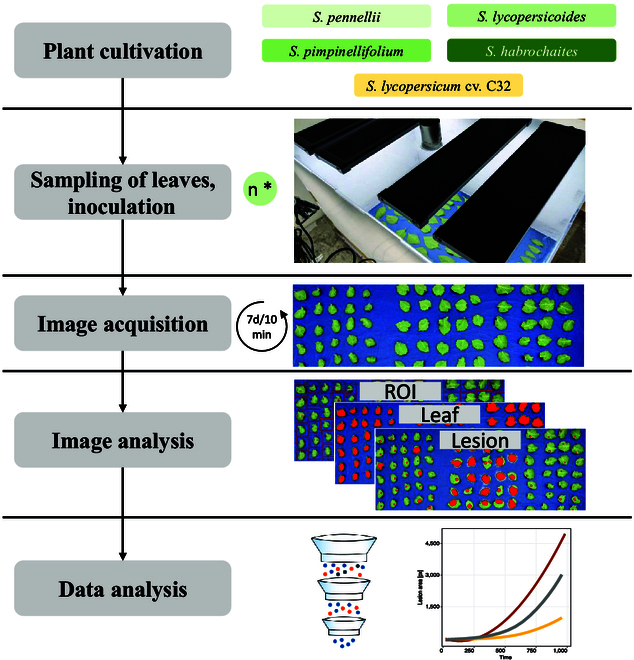
Overview of the high-throughput phenotyping assay. We cultivated 36 accessions from 4 wild tomato species (*S. pennellii, S. habrochaites, S, pimpinellifolium*, and *S. lycopersicoides*) and the domestic tomato cultivar “C32” in a greenhouse for detached-leaf assays. We harvested single leaves, inoculated using a *Sclerotinia sclerotiorum* mycelial suspension and placed them in the phenotyping boxes. Every 10 min, we captured images of the leaves for the duration of 1 week. We then manually defined regions of interest for each leaf and set thresholds for the feature classes “leaf”, “background”, and “lesion” in the HSV color scheme. Finally, we performed image analysis on all images, collected and filtered the data, and conducted further analytical steps to quantify the lesion development over time.

We screened multiple accessions of 4 wild tomato species (*S. pennellii, S. lycopersicoides, S. habrochaites,* and *S. lycopersicoides*) with a detached-leaf assay. All accessions of the same species were tested as one batch for up to 5 independent repetitions. To facilitate comparability between batches, *S. lycopersicum* cv. C32 was used as a control in every experiment. A schematic of the experimental procedures is displayed in Fig. 1.

### *S. sclerotiorum* inoculum preparation

For inoculation experiments, the *S. sclerotiorum* isolate 1980 or the OAH1:GFP (green fluorescent protein) isolate (for microscopic analysis only, [61]) was used. The fungus was alternatingly cultivated on potato dextrose agar (Sigma-Aldrich) and solid malic acid medium [62] at approximately 25 °C in the dark. Four 1-cm pieces of *S. sclerotiorum* inoculum were used to inoculate 100 ml of potato dextrose broth. After 4 days of incubation on a rotary shaker (24 °C, 120 rpm), a fungal mycelium suspension was generated: for this, the medium was mixed 2 times using a dispenser (IKA T25) for 10 s at 24,000 rpm. The mixture was then vacuum-filtrated through cheesecloth, and the remaining liquid was concentrated to an optical density (OD) of 1. For the negative control, fungal tissue was removed from the solution by centrifugation, and the supernatant was autoclaved. Tween 20 was used as a surfactant. Per leaf, one drop (10 μl) of inoculum was used.

### Plant growing conditions

**Fig. 2.**
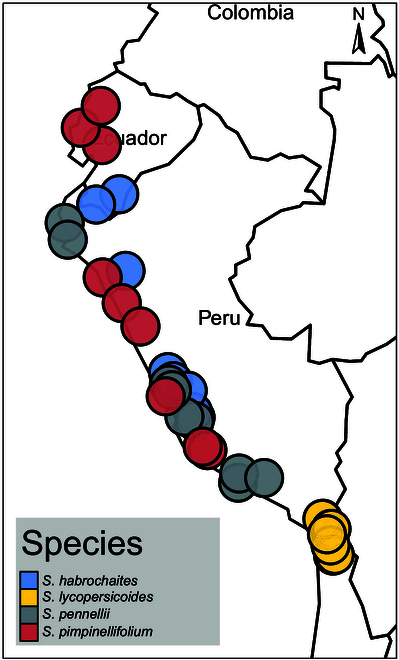
Sampling localities of wild tomato accessions used in this study. Seed material of all wild tomato accessions was provided from C. M. Rick Tomato Genetics Resource Center of the University of California, Davis (TGRC UC-Davis, http://tgrc.ucdavis.edu/). Individual dots represent the geographical origin of each accession.

Wild tomato germplasm was obtained from the C. M. Rick Tomato Genetics Resource Center of the University of California, Davis (TGRC UC-Davis, http://tgrc.ucdavis.edu/) (see Table S5). The species were selected to include genetically diverse species within the section Lycopersicon and a species from the section Lycopersicoides (Fig. 2). All plants were grown at the greenhouse facility of the Department of Phytopathology and Crop Protection, Institute of Phytopathology, Faculty of Agricultural and Nutritional Sciences, Christian Albrechts University, Kiel, Germany. Following seed surface sterilization using 2.75% hypochlorite (15 min incubation followed by washing twice with dH_2_O), seeds were sown in the substrate (STENDER C700, Germany) and cultivated in a growth chamber (21 °C, 65% rH, 16 h of 450 photosynthetically active radiation [PAR]). From the 3-leaf stage on, plants were cultivated in standard greenhouse conditions with supplement light (approximately 16 h/day, 15 to 25 °C at 50% to 70% rH). Plants were fertilized via the irrigation system (monthly, 1% Sagaphos Blue, Germany). Plants were propagated using cuttings (Chryzotop Grün 0.25%) and regularly screened for virus infection.

### Detached leaf assay

Detached leaf assays were conducted to measure QDR of a diverse panel of wild Solanaceae plants. A custom phenotyping system was adapted [30]. A 50 cm × 70 cm PMMA tray was filled with 8 layers of blue tissue paper and flooded with 700 ml of sterile dH_2_O. Plant leaves were placed abaxial side up onto the tissue and inoculated with 10 μl of mock/*S. sclerotiorum* suspension. Next, the tray is covered with a custom hood. The boxes were placed inside a growth chamber (24 °C) and incubated for 7 days. The assay was independently repeated 5 times. We used a representative set of 3 experiments for all further analysis. We tested potential border effects on the resistance parameters and found no statistically significant impact of leaf position on the measured parameters. Consequently, the leaves were not spatially randomized on the tray.

### Phenotyping platform

High-resolution images were acquired using RGB cameras (Yealink UVC30) mounted on the box. Cameras were controlled using Raspberry Pi microcomputers or desktop PCs running headless Ubuntu22. A cron daemon launched the image-acquisition script every 10 min. Plant lights also briefly (1 s) illuminate during nighttime for image capture to enable images in the dark while maintaining circadian rhythm. This was achieved by using the “Shelly Plus Plug S” wifi plug (Fig. S1).

### Image analysis

We adapted the “navautron” software package (https://github.com/A02l01/Navautron). The image analysis involved manually defining regions of interest (ROIs) using ImageJ (ImageJ Version 1.530). Further, HSV thresholds were optimized individually per box. For this, “assess_noChl.py” was used, and an overlay was generated in Gimp (Version 2.10). Once binary masks represented the respective feature classes (leaf_healthy, leaf_diseased, and background), the whole dataset was evaluated using the “infest.py” script. Segmentation was iterated and classified pixel was counted. The analysis includes functions from the python3 (Version 3.11.4) libraries “numpy” (Version 1.25.2), “opencv” (Version 4.8.1.78), “plantcv” (Version 4.0.1), and “scikit-image” (Version 0.22.0). The plantcv function “dilate” was used to remove leaf edges containing shadows with ksize = 9, *i* = 1 [63]. To improve thresholding accuracy (e.g., filling holes) on the lesion, an index filter was applied [ndimage.generic_filter(mask, threshold, size = 3, mode = “constant”)] with a condition to overwrite pixels deviating from the value of the majority of the surrounding pixels. np.sum(mask) was used to quantify the number of pixels in each feature class (lesion and leaf). Code and scripts can be found at https://github.com/seveein/QDR_Wild_Tomatoes.

### Microscopy analysis

Plant leaves were harvested and inoculated under standard conditions as described before but with either a GFP-expressing *S. sclerotiorum* strain, the *S. sclerotiorum* wild-type 1980, or the mock suspension. The leaves were evaluated at 12-h intervals using a Zeiss Discovery V20 stereomicroscope under bright light and fluorescent illumination (Zeiss HXP120). Images were taken using an AxioCam MRc camera.

### Statistical analysis

An interactive R-script (R-Version 4.3.2, R-Studio 2023.12.1+402) was utilized to extract lag-phase duration and lesion doubling time (LDT) to quantify resistance characteristics [30]. Each leaf’s lesion size over time was fitted against a 4-degree polynomial regression. The fit to the measured data point was reviewed for each sample. LDT and lag phase were determined based on a segmented regression analysis, expecting 2 linear phases: first, a linear phase during the lag period (no symptom development), and second, a linear log growth (symptom) during the exponential growth phase. The period prior to the lesion growth (LDT) is considered the lag phase, while the LDT represents the log(slope) of the linear growing curve in this area.

A 2-tier filtering pipeline was developed to increase accuracy and remove artifacts from the dataset. First, time points with leaf-size outliers were trimmed by removing 2.5% of the individual reads. Next, individual leaves with unexpectedly high variability in leaf area were excluded from the dataset. Therefore, samples with sd(leaf) > 10% of the mean(leaf) were removed from the dataset using a simple tidyverse (v. 2.0.0) pipeline.

As a measure of symptom development over time, the area under the disease progress curve (AUDPC) was calculated using the R-package agricolae (v. 1.3-6). General statistical analysis and visualization were conducted in RStudio (R-Version 4.3.2, R-Studio 2023.12.1+402 [64]) and the packages tidyverse [65], ggplot2 [66], ggpubr [67], and agricolae [68]. AUDPC is defined with *i* = time and *y_i_* = symptom severity at time = *i* as [68]:

$$\text{AUDPC}=\sum_{i=1}^{N}\frac{\left(y_{i}+y_{i+1}\right)\times \left(i-1\right)}{2}$$ (1)

For continuous variables (lag-phase duration, LDT, AUDPC, and tt100), a statistical model based on a generalized least squares model was defined [69]. In contrast, a generalized linear model was defined for binomial values (IF, 100%/*f*) [70]. These models included genotype and start date (without interaction effect).

The residuals corresponding to the continuous values were assumed to be approximately normally distributed and heteroscedastic concerning the different genotypes. These assumptions are based on a graphical residual analysis (Figs. S8 and S9). Based on these models, a pseudo *R*^2^ was calculated [71], and an analysis of variance (ANOVA) was conducted, followed by multiple contrast tests [72,73]. User-defined contrast matrices were used (a) to compare the species’ means with each other and (b) to compare the population means within their specific species with the corresponding species’ mean. The individual leaf area was previously found to have no significant influence on lesion area; therefore, it was not included in our statistical model [74]. A linear mixed-effects model was used to determine the relationship between AUDPC and predictors such as genotype, lag-phase duration, and LDT. Random intercepts were specified per start date to account for experimental repetitions.

Based on this model, fixed-effect values were extracted and used to predict AUDPC per genotype_i=1,2,3_ in relation to varying lag and LDT values.

$$\begin{array}{c} {\text{AUDPC}}_{i}={\text{Intercept}}_{i}+{\text{Coefficient}}_{{\mathrm{lag}}_{i}}\times \text{lag}+\\ {\text{Coefficient}}_{{\text{LDT}}_{i}}\times \text{LDT}+\\ {\text{Coefficient}}_{\text{la}g_{i}\times {\text{LDT}}_{i}}\times \text{lag}\times \text{LDT} \end{array}$$ (2)

The associated R-codes can be found at https://github.com/seveein/phenotyping_QDR_Wild_Tomatoes.

## Results

### Wild tomato species carry different levels of quantitative resistance against *S. sclerotiorum* depending on defense parameters

**Fig. 3.**
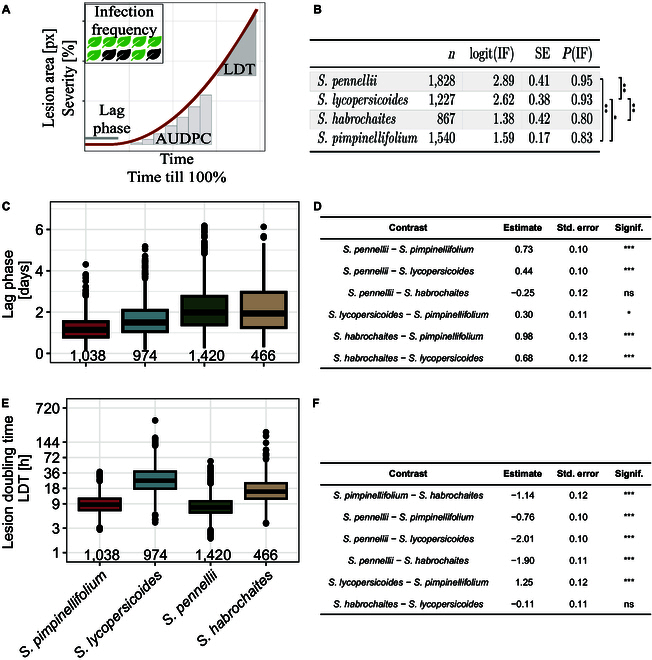
Wild tomato species possess a broad diversity of resistance against *S. sclerotiorum.* (A) Exemplary illustration of different QDR parameters used in this study. The infection frequency is defined as the percentage of leaves showing a lesion after 7 days of incubation. The lag phase is defined as the time till first visual symptoms appear. We used a segmented regression analysis to determine the lag phase’s end mathematically. The absolute lesion size is represented as pixel counts, whereas normalization against leaf area results in symptom severity. The area under the disease progress curve (AUDPC) is defined as the integral area under the severity curve, which depicts the severity over time. As a measure of the lesion spread, the lesion doubling time (LDT) describes the time till a lesion doubles its size. The time till a lesion covers 100% of a leaf is described by tt100%. (B) The infection frequency of *S. sclerotiorum* inoculum differs significantly between the host species. The table shows a meta-analysis of pooled accessions collected from 3 independent experiments. (C) Time till lesion formation (in days). The number on the *x*-axis indicates the count of individual leaves tested. (D) Statistical analysis of pairwise differences in lag-phase duration between the tested wild tomato species. Values are displayed in days and derived from a generalized least squares model. (E) Lesion growth rate during the exponential growth phase hours, plotted on a log scale. The number on the *x*-axis indicates the count of individual leaves tested. Raw values are plotted. (F) Statistical analysis of pairwise differences between the tested wild tomato species regarding lesion doubling time. Values are displayed as log(LDT[h]). Levels of significance are displayed as ***P < 0.001, **P < 0.01. *P < 0.05, *P* < 0.1.

We investigated the phenotypic diversity in QDR in 4 wild tomato species (*S. habrochaites, S. lycopersicoides, S. pennellii,* and *S. pimpinellifolium*) against the *S. sclerotiorum* isolate 1980 [13]. We used the “Navautron” automated phenotyping system for continuous image acquisition and applied a threshold-based segmentation algorithm to extract phenotypic data (Fig. S1). Hence, we calculated different QDR parameters such as IF, lag-phase duration, LDT, or AUDPC to quantify temporal dynamics of infection (Fig. 3). High variability between experimental runs with wild tomatoes has been described before [6,49,74]. To account for this, we applied a generalized least squares model (gls, continuous variables) and a generalized linear model (glm, discrete variables) for statistical analysis [69]. Overall, we discovered a great diversity of resistance phenotypes among the tested plant species. We found no 100% resistant accessions (Fig. S4). We observed a significant difference in lag-phase duration among plant species, which we define as the time from infection until the first symptoms appear (see Fig. 3A, C, and D). For instance, *S. pimpinellifolium* showed the shortest time from inoculation until lesion development (adjusted mean = 36.2 h). In contrast, *S. habrochaites* and *S. pennellii* displayed a significantly prolonged lag phase (both approximately 59 h) (see Table S1). Using segmented regression analysis, we determined the speed of lesion growth on individual leaves of the panel. The fastest-growing lesions were found on the species *S. pimpinellifolium* and *S. pennellii.* Lesions on *S. pennellii* and *S. pimpinellifolium* leaves doubled in size within approximately 11 h [6.56 log(LDT) and 6.55 log(LDT), respectively], while lesions on *S. habrochaites* and *S. lycopersicoides* spread significantly slower. Those lesions expanded with an average rate of approximately 7.7 log(LDT), corresponding to roughly 36 h (*S. habrochaites*) and 41 h (*S. lycopersicoides*) (see Table S2). Moreover, we observed that the success of disease establishment (IF) depends highly on the host species. We identified a significantly lower infection rate on *S. habrochaites* (corrected IF estimate 80 %), whereas *S. lycopersicoides* and *S. pennellii* displayed significantly higher IF (~93% and 95%, respectively) (Fig. 3B).

### Individual QDR measures show different levels of intraspecific variation and conservation on *S. pennellii* and *S. lycopersicoides* accessions

**Fig. 4.**
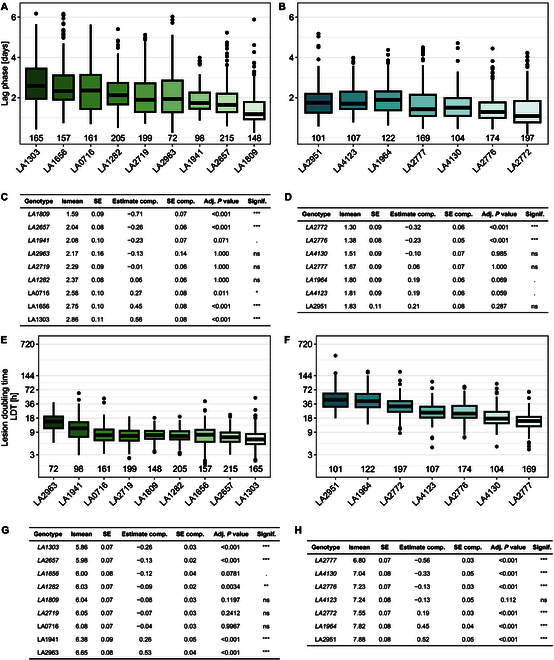
QDR parameters show different levels of variation depending on the host species (*S. pennellii and S. lycopersicoides*)*.* (A) The lag-phase duration (in days after infection) of *S. sclerotiorum* infection on *S. pennellii* accessions displays a higher level of intraspecific diversity than on accessions of *S. lycopersicoides* (B). (C and D) Variation statistics of the lag-phase duration contrasting each accession with the grand mean per species [*S. pennellii* (C); *S. lycopersicoides* (D)]. Estimates are displayed in days after inoculation. (E and F) The lesion doubling time (in hours) of *S. sclerotiorum* infection on *S. pennellii* accessions is lower than on *S. lycopersicoides*. (G) Variation statistics of LDT on *S. pennellii* and (H) *S. lycopersicoides.* lsmean values and SE indicate the adjusted mean and SE per population. Estimate comp., SE comp, and *P* values describe pairwise statistics of each accession against the grand mean. The numbers on the *x*-axis in panels A, B, E, and F indicate the count of individual leaves tested. Levels of significance are displayed as ***P < 0.001, **P < 0.01. *P < 0.05, *P* < 0.1.

To assess the within-species diversity of QDR phenotypes, we tested different accessions of each represented species. We collected phenotypic data from 7 *S. lycopersicoides* and 9 *S. pennellii* populations (Fig. 4), as well as 8 populations of *S. habrochaites* and 10 populations of the species *S. pimpinellifolium* (Figs. S2 and S3)*.* In particular, the comparison of *S. lycopersicoides* and *S. pennellii* highlights that QDR diversity differs between species. We observed that the (adjusted) mean duration of the lag phase on different *S. pennellii* accessions ranged from 1.59 days (38 h, LA1809) to 2.86 days (68 h, LA1303) (Fig. 4A and C). Using a generalized least squares model, we identified accessions with a significantly shorter lag phase than the grand mean of the species (LA1809 and LA2657). In contrast, the accessions LA1656 and LA1303 displayed a significantly longer lag phase (2.75 days [66 h] and 2.86 days [68 h], respectively) (Fig. 4A and C). Next, we observed a significantly shorter overall lag-phase duration of *S. lycopersicoides* accessions than *S. pennellii.* Accordingly, the first symptoms appeared after 1.3 days (31 h, LA2772) and the latest appeared at 1.83 days after inoculation (43 h, LA1966). The overall time till initial symptom development was more conserved; only 2 *S. lycopersicoides* accessions deviated significantly from the grand mean, being more susceptible than the overall species level (LA2776 and LA2772) (Fig. 4B and D). Similarly, we found a lack of variation in lag-phase duration in the populations of *S. pimpinellifolium*. At the same time, *S. habrochaites* accessions displayed a wider variability of lag-phase phenotypes (Fig. S2 and Table S3).

Next, we analyzed the variability of the lesion growth rate between accessions of each species using the logarithmic LDT. We observed that all tested *S. pennellii* accessions displayed an average LDT ranging from 5.84 h (LA1303) to 13.07 h (LA2963). Five accessions (LA1809, LA1282, LA2719, LA2657, and LA1303) have a significantly faster lesion development than the grand mean (LDT < 11 h). The populations LA2963 and LA1941 displayed a significantly longer LDT (13.07 and 9.8 h, respectively) (Fig. 4E and G). Generally, we found that symptoms of *S. lycopersicoides* grew significantly slower (observed range: 14.9 h to 40 h). However, we still observed a significant within-species variability. For instance, symptoms on leaves of the accession LA2951 doubled within lsmean = 7.88 log(LDT) (approximately 44 h), while lesions of LA2777 expanded much faster at lsmean = 6.8 log(LDT) (15 h, Fig. 4F and H). We observed a high variability among the accessions for *S. pennellii* and *S. lycopersicoides*, mostly deviating from the species mean in LDT with high significance. Interestingly, the LDT on *S. habrochaites* and *S. pimpinellifolium* appeared much more conserved between the accessions, as only a few samples significantly differed from the grand mean (Fig. S3 and Table S4).

### Disease resistance measures are not linked and characterize distinct components of QDR

**Fig. 5.**
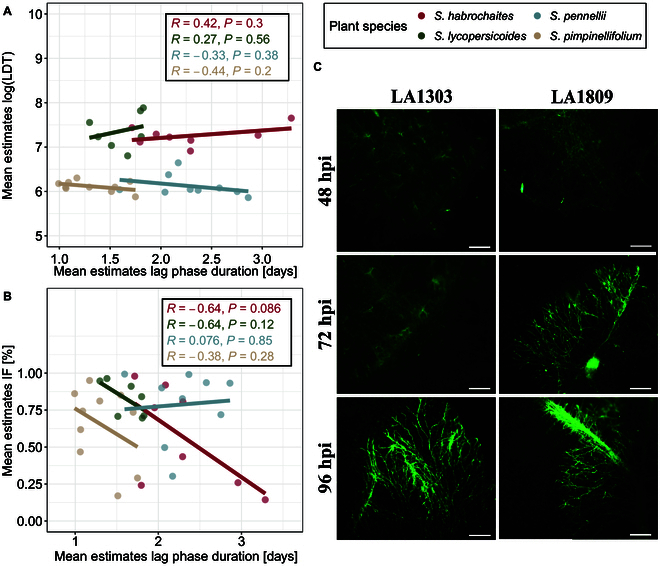
Different QDR parameters are independent from each other. (A) Pearson correlation analysis of LDT and lag-phase duration. Dots represent the least squares of each accession. (B) Pearson correlation analysis between the infection frequency and lag-phase duration. Dots represent infection frequency adjusted per-accession estimates from glm/gls. (C) The lag-phase duration of *Ss1980:GFP* infection on *S. pennellii* genotypes is reflected in fungal growth dynamics. Images show a representative selection of 10 biological replicates. The scale bar indicates 500 μm.

To test whether fungal infection is directly linked to delayed lesion growth, we conducted microscopy assays using a GFP-tagged *S. sclerotiorum* mutant of the *S. sclerotiorum* isolate 1980 [75]. We selected 2 accessions from *S. pennellii* with significantly altered lag-phase duration. At 72 h past inoculation (hpi), freshly developed mycelium was observed on leaves of the *S. pennellii* accession with the shortest lag-phase duration (LA1809). In contrast, on the less susceptible accession LA1303, the first fungal structures started growing at 96 hpi (Fig. S7). Fluorescent microscopy imaging showed that fungal mycelial structures were always accompanied by clear formation of necrotic lesions but cannot be observed prior to visual lesion development (Fig. 5C and Fig. S7). Thus, this shows that a longer lag phase does not represent any latent or biotrophic infection and that IF and lag-phase duration are likely uncoupled phenomena.

We performed a correlation analysis to consolidate the relationship between the QDR parameters further. First, we tested the overall relation of lsmean LDT and lsmean lag-phase duration by pooling all accessions of all species. We found that LDT and lag phase were independent (*R* = 0.14), with no significant relationship (*P* = 0.42) (Fig. S5). We also tested the correlation between QDR mechanisms at the species level. We found only minor linear relationships between LDT and lag phase for the 4 tested species. However, we found a weak, significant negative correlation between IF and the duration of the lag phase (lsmean) in *S. habrochaites* (*R* = −0.64, *p* = 0.086) (Fig. 5A and B). For the remaining species, no significant correlation was found. We did not find a single host accession with high levels of resistance in both LDT and lag-phase duration.

### Severity analysis reveals distinct resistance phenotypes against *S. sclerotiorum* within a single species

**Fig. 6.**
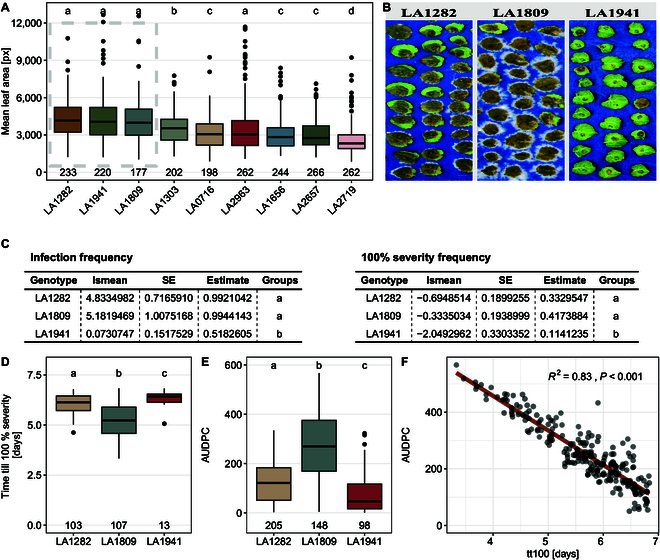
*S. pennellii* accession LA1941 harbors a significantly elevated level of quantitative resistance against *S. sclerotiorum.* (A) Mean leaf area of *S. pennellii* accessions quantified during infection experiments. The data of 3 independent experiments are shown. HSD test was performed to identify cluster with similar leaf size. Selected plants with similar leaf size are indicated by the box. (B) Exemplary images of *S. sclerotiorum* infections on the *S. pennellii* populations LA1282, LA1809, and LA1941 at 7 days post-inoculation. (C) Statistical analysis of infection frequency (IF) and frequency of fully infected leaves at the end of experiment. “lsmean” represents the estimate as logits, while “estimate” represents the estimated probability. (D) Comparison of time till lesion saturation of *S. sclerotiorum* on *S. pennellii* genotypes. (E) Area under disease progress curve (AUDPC) of 3 *S. pennellii* populations with similar leaf size. Wilcoxon test was performed for levels of significance. Time series data from previous experiments was used. (F) Pearson correlation analysis of tt100 vs. AUDPC. The numbers at the base of all boxplots represent individual leaves tested. All statistics were calculated using a glm/gls with custom contrast matrizes. Compact letter displays were determined using the package “multcompLetters” with a threshold of *P* < 0.05.

For an in-depth analysis of disease severity, we selected 3 *S. pennellii* accessions with similar leaf sizes: LA1282, LA1809, and LA1941 (Fig. 6A). While symptoms developed on most of the leaves, the impact of infection is highly dependent on the respective accession (see Fig. 6B). Accession LA1941 shows a significantly lower IF (~51%) and a significantly lower rate of fully infected leaves than LA1809 (approximately 11% vs. approximately 41%) or LA1282 (approximately 33%, Fig. 6C).

**Fig. 7.**
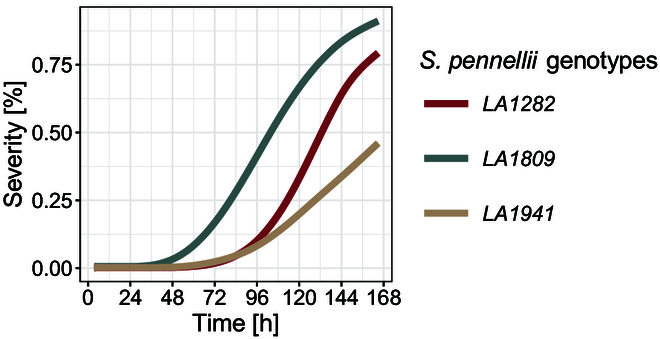
Exemplary growth curve of 3 *S. pennellii* accessions with different resistance levels against *S. sclerotiorum.* Shown is the mean symptom severity of each accession as share of leaf area over the period of 7 days. The experiment was independently repeated 3 times. *n*_LA1282_ = 205, *n*_LA1809_ = 148, *n*_LA941_ = 98.

We further found differences regarding the speed of lesion growth over time between the genotypes. While lesions on LA1282 and LA1941 reached 100% severity within 6.5 to 7 days, we observed that symptoms on LA1809 reached the point of saturation significantly faster (after approximately 5 days, Fig. 7). This is also reflected by significantly increased AUDPC values of LA1809 (AUDPC approximately 250). In LA1282, we measured not only a lower AUDPC compared to LA1809 (Fig. 6E), but also an insignificant difference in the rate of fully infected leaves (Fig. 6C). This could hint toward a delayed but explosive lesion growth on LA1282 (Fig. 7).

### The moderation of QDR parameters is genotype-dependent

**Fig. 8.**
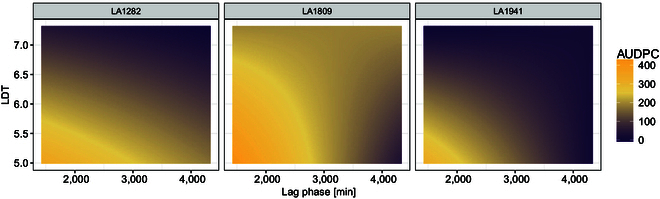
The crosstalk of lag-phase duration and LDT is highly genotype-dependent and specifically determines the symptom severity. We used the *S. pennellii* accessions LA1282, LA1809, and LA1941 to test for the genotype-dependent relationship between lag and LDT. Therefore, we extracted the estimates for the factors LDT and lag per each genotype from an ANOVA based on a generalized least square model (Table 1). The per-genotype AUDPC was modeled using the extracted estimates over a range of values representing the plausible range of lag/LDT values. Crosses represent the observed mean AUDPC (Fig. 6E).

Next, we used a linear mixed-effect model (lme) to test which of the factors have the strongest effects on disease severity on those accessions (*S. pennellii* LA1282, LA1809, and LA1941). Following the ANOVA, we found a significant influence of most tested variables (genotype, lag phase, and LDT) on the AUDPC (Table). Strikingly, we found that the genomic background of the tested plants is insufficient to explain the observed diversity in AUDPC. In other words, we observe a significant relationship between lag, LDT, and their interaction with the genotype. Because of this, we extracted the fixed-effect estimates from the lme and generated predictor functions for the AUDPC of each genotype. Then, we modeled the AUDPC using high-confidence lag and LDT values from previous observations (see Fig. 3C and D). We observed the highly variable influence of lag-phase duration, LDT, and their interaction on the AUDPC (Fig. 8). Strikingly, we found that variation of the LDT has almost no influence on the AUDPC of LA1809 besides the generally elevated severity level (Fig. 8). Further, we found that only a prolonged lag-phase duration might contribute to an increased potential for lower severity in LA1809 (Fig. 8). However, the influence of longer lag phase is reduced with increasing LDT. For leaves of the accessions LA1282 and LA1941, we found a stronger combined effect of lag-phase and LDT on the severity. More specifically, a prolonged lag phase might lead to a small reduction of the symptom severity on LA1282 while reducing the AUDPC on LA1941 more rapidly. Further, we observe that a prolonged LDT reduced symptom severity in both LA1282 and LA1941.

**Table.** Statistical analysis of the effects of genotype, lag-phase duration, LDT, and their interactions on disease severity (AUDPC) of the *S. pennellii* accessions LA1282, LA1809, and LA1941. Results of an analysis of variance (ANOVA) based on a linear mixed-effects model are shown.

	numDF	denDF	*F* value	*P* value
Intercept	1	437	136.8	<0.001
Genotype	2	437	211.34	<0.001
Lag	1	437	251.68	<0.001
LDT	1	437	90.41	<0.001
Genotype:Lag	2	437	8.54	<0.001
Genotype:LDT	2	437	2.32	0.099
Lag:LDT	1	437	21.91	<0.001
Genotype:Lag:LDT	2	437	3.3	0.038

## Discussion

### QDR against *S. sclerotiorum* is highly diverse in *Solanum* spp.

Wild tomato species have been screened for quantitative resistance phenotypes against many diseases, including Tomato brown rugose fruit virus*, Phytophthora infestans, Alternaria solani, Fusarium* spp., or *B. cinerea* [8,49,50,74,76–79]. However, high hurdles in characterizing QDR on a phenotypic level limit detailed insights into the functional role of QDR against necrotrophic pathogens. This was mostly due to the lack of affordable high-throughput phenotyping facilities [1,2,5,8,80]. Here, we present a unique dataset of high-resolution QDR phenotypes against *S. sclerotiorum* on a diverse set of wild *Solanum* species derived from a low-budget phenotyping setup, using detached leaf infections. There are potential disadvantages to using detached leaf assays, i.e., the lack of feedback mechanisms between leaf and root/shoot, which could make plants more susceptible if this feedback plays a role in amplifying the defense signal, or more susceptible if this feedback provides susceptibility factors or nutrients for the pathogen. The immense gain in efficiency in both experimental setup and data analysis, together with the fact that detached leaf assays have been used successfully in tomato and with *S. sclerotiorum* and other necrotrophic leaf pathogens, justifies the decision to develop our methods for detached leaves [30,49,50,74,78]. In total, we tested almost 7,000 leaves over the duration of 7 days with approximately 1,000 measurements each, resulting in approximately 7 million data points. We used this unique dataset to characterize the lesion development of infected leaves and applied advanced statistical analysis methods to extract more specific descriptors for QDR, such as lag phase, LDT, or AUDPC [30]. Because of this system’s scale and temporal resolution, we generated novel insights into the phenomena contributing to QDR.

### Interspecific QDR phenotypes follow a wide distribution

As expected, we observed a diverse range of disease phenotypes, as demonstrated in previous studies [6,49,78]. None of the tested accessions carried complete resistance against *S. sclerotiorum*, although we found a wide distribution of infection phenotypes. Also, no high “universal” level of partial resistance or tolerance among multiple QDR parameters was found, as none of the species harbors significant advantages in multiple measures (IF, lag phase, or LDT). Complete resistance against *S. sclerotiorum* is rarely found in cultivated crops [32,52,60,81]. We provide evidence that the time till the emergence of the first lesions (lag phase) is highly variable within and between host species, with only *S. lycopersicoides* showing a rather conserved lag-phase duration (Fig. 4B). Interestingly, Barbacci et al. [30] reported that in *Arabidopsis thaliana*, the lag-phase duration is mostly influenced by the *S. sclerotiorum* isolate rather than the host accession. The comparably low genetic diversity of the host may have influenced the observed range of QDRs. Standing genetic variation is considered much higher in (predominantly) outcrossing *Solanum* species than in inbreeding *A. thaliana* accessions [82]*.* Accordingly, we assume that the influence of genetic features on the lag-phase duration depends on the specific genomic background of the host plant species. However, fungal influences on pathogenesis cannot be ignored, as the concept of the “extended phenotype”, describing the interaction of both genomes, i.e., a genotype × genotype (G × G) interaction for host and pathogen, for one phenotype, is well established [37,83]. Furthermore, quantitative host resistance features have been described to interact with the pathogen’s genotype as described for camalexin-associated resistance [50,84].

### QDR phenotypes also differ on the intraspecific level but at varying degrees

High variability of QDR phenotypes among genotypes of the same plant species has been reported on multiple hosts before [6,30,49,74,85,86]. We show that the degree of variability depends on the host species and the respective resistance parameter. Whereas LDT is rather stable among *S. pimpinellifolium* accessions, it is highly variable on *S. lycopersicoides* accessions (see Fig. 4 and Fig. S3). The specific forms of QDR phenotypes might hint at independent regulatory mechanisms and different evolutionary backgrounds with relatively recent developments, leading to genetic variation, rather than conserved QDR mechanisms. Host adaptation to natural habitats and its influence on disease resistance has been studied before [87,88]. Adaptation might explain disease phenotypes as most *S. lycopersicoides* accessions show significantly prolonged LDT. The habitat of *S. lycopersicoides* faces much more rain than the other species, leading to higher chances of successful infection events than in relatively dry habitats, thus requiring mechanisms to fight established infections. In contrast, drought-resistant *S. pennellii* has high capabilities in delaying infection events, while it lacks defense efficacy once an infection is established (Fig. 3), similar to the *S. chilense* desert population losing resistance against the fungus *Passalora fulva* [46,87,89]. However, to truly test these hypotheses, significantly higher sample sizes and infections under natural conditions would be required, possibly paired with screenings of the morphological properties of the species to assess the pleiotropic influence of habitat adaptation on QDR, e.g., via cuticle thickness or stomata density.

### QDR and genotype × genotype × environment interactions

*S. pennellii* accession LA0716 was characterized as relatively resistant against *B. cinerea,* while this genotype is highly susceptible to *S. sclerotiorum* (Fig. S6) [6]. Also in *S. chilense*, QDR phenotypes vary between the pathogen, suggesting the presence of pathogen-specific regulatory mechanisms [78]. However, the pathogen diversity tested in such studies might greatly affect the observed degree of resistance. A study with *Phytophthora infestans* on 85 *S. chilense* accessions showed that the relative differences in resistance phenotypes between individuals were mainly determined by the plant genotype, with modest effects of pathogen isolate used [49]. In contrast, large-scale screenings of infections with different *B. cinerea* isolates showed a clear genotype × genotype (G × G) effect both on panels of wild and domesticated tomatoes and on *A. thaliana* [37,50,74,90,91]. In addition, we have shown in *S. chilense* that QDR phenotypes, like the IF, can be correlated with the phytohormone ethylene [92]. Knowing that such phytohormonal regulation is also affected by abiotic, environmental (E) factors like temperature, humidity, and light availability, we propose that QDR polymorphism is implemented in a complex signaling network affected by G × G × E interactions [1,5,93,94].

### QDR is determined by the interplay of QDR mechanisms

QDR is commonly defined as a highly interconnected regulatory network with an integrated, pleiotropic role in general plant metabolism [1]. Therefore, the linkage of different defense mechanisms, like IF and lag-phase duration, could be a good perspective for resistance breeding. However, we did not observe strong correlations between QDR parameters and did not find a species or accession with a universal high resistance level for all tested parameters. Disconnected QDR parameters have been reported before: *Xanthomonas axanopodis* mutants showed increased IF but a reduced lesion growth rate on cassava and *B. cinerea* showed unconnected IF and lesion expansion rates on wild tomatoes [6,95]*.* We used the presented phenotyping platform to show that the moderation or cross-talk between defense mechanisms is genotype-specific and differs even between accessions of the same species (Fig. 8). Based on these findings, we propose a model for QDR against necrotrophic pathogens involving 3 genetically distinct mechanisms: (a) prohibition of initial infection, (b) retardation of disease outbreaks, and (c) deceleration of ongoing infections.

### Disease severity is specifically determined by genotype-dependent moderation of QDR mechanisms

We used 3 differently severely infected *S. pennellii* genotypes to describe the influence of 2 of the QDR mechanisms (retardation and deceleration of symptom development) on overall symptom severity. Interestingly, the different accessions possess diverse capabilities in moderating the QDR mechanisms, as our model-based approach indicates contrasting roles of LDT and lag-phase duration. In *A. thaliana,* it was shown that lesion traits, like lesion size or shape, are also controlled by genetically distinct mechanisms [90]. Previous work showed that defense-associated hormone responses greatly differ between different wild tomato accessions and even within the same population. In *S. chilense,* ethylene responses could only be linked to IF in one population but not in others [92]. Therefore, we argue that the orchestration of QDR measures highly depends on the specific genetic background, and future studies should determine the complex interplay between various QDR-regulating mechanisms [94].

In this study, we used a new phenotyping platform to derive different QDR-related phenotypes. The low cost and high flexibility of the system allowed us to screen a big set of diverse plants relatively fast, and therefore, we identified new genotypes with distinct QDR properties. Accordingly, we characterized accessions and species with beneficial properties as significantly longer lag-phase duration (*S. pennellii,* LA1303 and LA1656) or prolonged LDT (*S. lycopersicoides,* LA2951 and LA1964). Accordingly, we suggest that *S. pennellii* accessions are specialized in delaying lesion development, whereas *S. lycopersicoides* accessions are more capable of slowing down the spread of established lesions. Follow-up research is needed to identify the genes underlying these differences. Moreover, the influence of increasing lack of nutrition during the time of the experiment must be critically evaluated to exclude starvation-induced loss of resistance. The resolution of the present dataset will enhance the ability to predict distinct defense phases, facilitating more targeted sampling mechanisms for transcriptomic or metabolomic analysis. This can help breed durable resistance in tomato crops with delayed and less severe symptoms without inducing strong evolutionary pressure. The sustainability of major R-gene-mediated resistance (including pyramiding of such) has regularly been questioned [4,96]. Facilitating the concept of QDR is proposed to thwart the arms race between plant hosts and pathogens. QDR phenotypes specifically tolerate disease to a certain extent without applying a strong bottleneck onto the pathogen population [14]. Our findings provide major insights into the architecture of QDR mechanisms and will help in the targeted functional characterization of QDR. By disentangling end-point QDR phenotypes into discrete resistance mechanisms, the functional characterization of genetic features controlling QDR will become much more targeted. Based on this study, the factors influencing the level of QDR can be explained in much more detail.

## Data Availability

Additional data can be found in the Supplementary Materials. All scripts used for this study as well as input data are available at https://github.com/seveein/phenotyping_QDR_Wild_Tomatoes.

## References

[1] Poland JA, Balint-Kurti PJ, Wisser RJ, Pratt RC, Nelson RJ. Shades of gray: The world of quantitative disease resistance. Trends Plant Sci. 2009;14(1):21–29. 19062327 10.1016/j.tplants.2008.10.006

[2] Roux F, Voisin D, Badet T, Balagué C, Barlet X, Huard-Chauveau C, Roby D, Raffaele S. Resistance to phytopathogens *e tutti quanti*: Placing plant quantitative disease resistance on the map: Quantitative disease resistance in plants. Mol Plant Pathol. 2014;15(5):427–432. 24796392 10.1111/mpp.12138 PMC6638617

[3] Mbengue M, Navaud O, Peyraud R, Barascud M, Badet T, Vincent R, Barbacci A, Raffaele S. Emerging trends in molecular interactions between plants and the broad host range fungal pathogens *Botrytis cinerea* and *Sclerotinia sclerotiorum*. Front Plant Sci. 2016;7:422. 27066056 10.3389/fpls.2016.00422 PMC4814483

[4] Brown JKM. Durable resistance of crops to disease: A Darwinian perspective. Annu Rev Phytopathol. 2015;53:513–539. 26077539 10.1146/annurev-phyto-102313-045914

[5] Gou M, Balint-Kurti P, Xu M, Yang Q. Quantitative disease resistance: Multifaceted players in plant defense. J Integr Plant Biol. 2023;65(2):594–610. 36448658 10.1111/jipb.13419

[6] ten Have A, van Berloo R, Lindhout P, van Kan JAL. Partial stem and leaf resistance against the fungal pathogen *Botrytis cinerea* in wild relatives of tomato. Eur J Plant Pathol. 2007;117:153–166.

[7] Tian L, Li J, Xu Y, Qiu Y, Zhang Y, Li X. A MAP kinase cascade broadly regulates the lifestyle of *Sclerotinia sclerotiorum* and can be targeted by HIGS for disease control. Plant J. 2023;118(2):324–344. 38149487 10.1111/tpj.16606

[8] Corwin JA, Kliebenstein DJ. Quantitative resistance: More than just perception of a pathogen. Plant Cell. 2017;29(4):655–665. 28302676 10.1105/tpc.16.00915 PMC5435431

[9] Boudhrioua C, Bastien M, Torkamaneh D, Belzile F. Genome-wide association mapping of *Sclerotinia sclerotiorum* resistance in soybean using whole-genome resequencing data. BMC Plant Biol. 2020;20(1):195. 32380949 10.1186/s12870-020-02401-8 PMC7333386

[10] Frey LA, Vleugels T, Ruttink T, Schubiger FX, Pégard M, Skøt L, Grieder C, Studer B, Roldán-Ruiz I, Kölliker R. Phenotypic variation and quantitative trait loci for resistance to southern anthracnose and clover rot in red clover. Theor Appl Genet. 2022;135(12):4337–4349. 36153770 10.1007/s00122-022-04223-8 PMC9734235

[11] Fusari CM, Di Rienzo JA, Troglia C, Nishinakamasu V, Moreno MV, Maringolo C, Quiroz F, Álvarez D, Escande A, Hopp E, et al. Association mapping in sunflower for sclerotinia head rot resistance. BMC Plant Biol. 2012;12:93. 22708963 10.1186/1471-2229-12-93 PMC3778846

[12] Wu J, Cai G, Tu J, Li L, Liu S, Luo X, Zhou L, Fan C, Zhou Y. Identification of QTLs for resistance to Sclerotinia stem rot and BnaC.IGMT5.A as a candidate gene of the major resistant QTL SRC6 in *Brassica napus*. PLoS One. 2013;8(7): Article e67740. 23844081 10.1371/journal.pone.0067740 PMC3699613

[13] Derbyshire M, Denton-Giles M, Hegedus D, Seifbarghy S, Rollins J, Van Kan J, Seidl MF, Faino L, Mbengue M, Navaud O, et al. The complete genome sequence of the phytopathogenic fungus *Sclerotinia sclerotiorum* reveals insights into the genome architecture of broad host range pathogens. Genome Biol Evol. 2017;9(3):593–618. 28204478 10.1093/gbe/evx030 PMC5381539

[14] Willocquet L, Savary S, Yuen J. Multiscale phenotyping and decision strategies in breeding for resistance. Trends Plant Sci. 2017;22(5):420–432. 28258957 10.1016/j.tplants.2017.01.009

[15] Dracatos PM, Lück S, Douchkov DK. Diversifying resistance mechanisms in cereal crops using microphenomics. Plant Phenomics. 2023;5:0023. 37040289 10.34133/plantphenomics.0023 PMC10076052

[16] Walsh JJ, Mangina E, Negrão S. Advancements in imaging sensors and AI for plant stress detection: A systematic literature review. Plant Phenomics. 2024;6:0153. 38435466 10.34133/plantphenomics.0153 PMC10905704

[17] Fahlgren N, Feldman M, Gehan MA, Wilson MS, Shyu C, Bryant DW, Hill ST, McEntee CJ, Warnasooriya SN, Kumar I, et al. A versatile phenotyping system and analytics platform reveals diverse temporal responses to water availability in *Setaria*. Mol Plant. 2015;8(10):1520–1535. 26099924 10.1016/j.molp.2015.06.005

[18] Watt M, Fiorani F, Usadel B, Rascher U, Muller O, Schurr U. Phenotyping: New windows into the plant for breeders. Annu Rev Plant Biol. 2020;71:689–712. 32097567 10.1146/annurev-arplant-042916-041124

[19] Bock CH, Barbedo JGA, Del Ponte EM, Bohnenkamp D, Mahlein A-K. From visual estimates to fully automated sensor-based measurements of plant disease severity: Status and challenges for improving accuracy. Phytopathol Res. 2020;2:9.

[20] Mahlein A-K. Plant disease detection by imaging sensors—Parallels and specific demands for precision agriculture and plant phenotyping. Plant Dis. 2016;100(2):241–251. 30694129 10.1094/PDIS-03-15-0340-FE

[21] Mahlein A-K, Kuska MT, Thomas S, Wahabzada M, Behmann J, Rascher U, Kersting K. Quantitative and qualitative phenotyping of disease resistance of crops by hyperspectral sensors: Seamless interlocking of phytopathology, sensors, and machine learning is needed! Curr Opin Plant Biol. 2019;50:156–162. 31387067 10.1016/j.pbi.2019.06.007

[22] Mutka AM, Bart RS. Image-based phenotyping of plant disease symptoms. Front Plant Sci. 2015;5:734. 25601871 10.3389/fpls.2014.00734 PMC4283508

[23] Simko I, Jimenez-Berni JA, Sirault XRR. Phenomic approaches and tools for phytopathologists. Phytopathology. 2017;107(1):6–17. 27618193 10.1094/PHYTO-02-16-0082-RVW

[24] Tanner F, Tonn S, de Wit J, Van den Ackerveken G, Berger B, Plett D. Sensor-based phenotyping of above-ground plant-pathogen interactions. Plant Methods. 2022;18(1):35. 35313920 10.1186/s13007-022-00853-7 PMC8935837

[25] Anim-Ayeko AO, Schillaci C, Lipani A. Automatic blight disease detection in potato (*Solanum tuberosum* L.) and tomato (*Solanum lycopersicum*, L. 1753) plants using deep learning. Smart Agric Technol. 2023;4:100178.

[26] Kuska MT, Heim RHJ, Geedicke I, Gold KM, Brugger A, Paulus S. Digital plant pathology: A foundation and guide to modern agriculture. J Plant Dis Prot. 2022;129(3):457–468. 10.1007/s41348-022-00600-z PMC9046714 35502325

[27] Tang Z, He X, Zhou G, Chen A, Wang Y, Li L, Hu Y. A precise image-based tomato leaf disease detection approach using PLPNet. Plant Phenomics. 2023;5:0042. 37228516 10.34133/plantphenomics.0042 PMC10204740

[28] Kersting K, Bauckhage C, Wahabzada M, Mahlein A-K, Steiner U, Oerke E-C, Römer C, Plümer L. Feeding the world with big data: Uncovering spectral characteristics and dynamics of stressed plants. In: Lässig J, Kersting K, Morik K, editors. *Computational sustainability*. Cham: Springer International Publishing; 2016. p. 99–120.

[29] Poorter H, Hummel GM, Nagel KA, Fiorani F, Von Gillhaussen P, Virnich O, Schurr U, Postma JA, Van De Zedde R, Wiese-Klinkenberg A. Pitfalls and potential of high-throughput plant phenotyping platforms. Front Plant Sci. 2023;14:1233794. 37680357 10.3389/fpls.2023.1233794 PMC10481964

[30] Barbacci A, Navaud O, Mbengue M, Barascud M, Godiard L, Khafif M, Lacaze A, Raffaele S. Rapid identification of an Arabidopsis NLR gene as a candidate conferring susceptibility to *Sclerotinia sclerotiorum* using time-resolved automated phenotyping. Plant J. 2020;103(2):903–917. 32170798 10.1111/tpj.14747 PMC7497225

[31] FAO. Agricultural production statistics 2000–2022. FAO. 2023. 10.4060/cc9205en

[32] Bolton MD, Thomma BPHJ, Nelson BD. *Sclerotinia sclerotiorum* (lib.) de Bary: Biology and molecular traits of a cosmopolitan pathogen. Mol Plant Pathol. 2006;7(1):1–16. 20507424 10.1111/j.1364-3703.2005.00316.x

[33] Foolad MR, Merk HL, Ashrafi H. Genetics, genomics and breeding of late blight and early blight resistance in tomato. Crit Rev Plant Sci. 2008;27(2):75–107.

[34] Schmey T, Tominello-Ramirez CS, Brune C, Stam R. *Alternaria* diseases on potato and tomato. Mol Plant Pathol. 2024;25(3): Article e13435. 38476108 10.1111/mpp.13435 PMC10933620

[35] Zalom FG. Pests, endangered pesticides and processing tomatoes. Acta Hortic. 2003;223–233.

[36] Einspanier S, Susanto T, Metz N, Wolters PJ, Vleeshouwers VGAA, Lankinen Å, Liljeroth E, Landschoot S, Ivanović Ž, Hückelhoven R, et al. Whole-genome sequencing elucidates the species-wide diversity and evolution of fungicide resistance in the early blight pathogen *Alternaria solani*. Evol Appl. 2022;15(10):1605–1620. 36330303 10.1111/eva.13350 PMC9624079

[37] Rowe HC, Kliebenstein DJ. All mold is not alike: The importance of intraspecific diversity in necrotrophic plant pathogens. PLoS Pathog. 2010;6(3): Article e1000759. 20361052 10.1371/journal.ppat.1000759 PMC2845657

[38] Schmey T, Small C, Einspanier S, Hoyoz LM, Ali T, Gamboa S, Mamani B, Sepulveda GC, Thines M, Stam R. Small-spored *Alternaria* spp. (section *Alternaria*) are common pathogens on wild tomato species. Environ Microbiol. 2023;25(10):1830–1846. 37171093 10.1111/1462-2920.16394

[39] Silva RA, Lehner MS, Paula Júnior TJ, Mizubuti ESG. Fungicide sensitivity of isolates of *Sclerotinia sclerotiorum* from different hosts and regions in Brazil and phenotypic instability of thiophanate-methyl resistant isolates. Trop Plant Pathol. 2024;49:93–103.

[40] Wang Q, Mao Y, Li S, Li T, Wang J, Zhou M, Duan Y. Molecular mechanism of *Sclerotinia sclerotiorum* resistance to succinate dehydrogenase inhibitor fungicides. J Agric Food Chem. 2022;70(23):7039–7048. 35666187 10.1021/acs.jafc.2c02056

[41] Bolger A, Scossa F, Bolger ME, Lanz C, Maumus F, Tohge T, Quesneville H, Alseekh S, Sørensen I, Lichtenstein G, et al. The genome of the stress-tolerant wild tomato species *Solanum pennellii*. Nat Genet. 2014;46(9):1034–1038. 25064008 10.1038/ng.3046 PMC7036041

[42] Rebetzke GJ, Jimenez-Berni J, Fischer RA, Deery DM, Smith DJ. Review: High-throughput phenotyping to enhance the use of crop genetic resources. Plant Sci. 2019;282:40–48. 31003610 10.1016/j.plantsci.2018.06.017

[43] Pease JB, Haak DC, Hahn MW, Moyle LC. Phylogenomics reveals three sources of adaptive variation during a rapid radiation. PLoS Biol. 2016;14(2): Article e1002379. 26871574 10.1371/journal.pbio.1002379 PMC4752443

[44] Böndel KB, Lainer H, Nosenko T, Mboup M, Tellier A, Stephan W. North–south colonization associated with local adaptation of the wild tomato species *Solanum chilense*. Mol Biol Evol. 2015;32(11):2932–2943. 26232423 10.1093/molbev/msv166

[45] Fischer I, Camus-Kulandaivelu L, Allal F, Stephan W. Adaptation to drought in two wild tomato species: The evolution of the *Asr* gene family. New Phytol. 2011;190(4):1032–1044. 21323928 10.1111/j.1469-8137.2011.03648.x

[46] Kahn TL, Fender SE, Bray EA, O’Connell MA. Characterization of expression of drought- and abscisic acid-regulated tomato genes in the drought-resistant species *Lycopersicon pennellii*. Plant Physiol. 1993;103(2):597–605. 12231965 10.1104/pp.103.2.597 PMC159020

[47] Nosenko T, Böndel KB, Kumpfmüller G, Stephan W. Adaptation to low temperatures in the wild tomato species *Solanum chilense*. Mol Ecol. 2016;25(12):2853–2869. 27037798 10.1111/mec.13637

[48] Stam R, Nosenko T, Hörger AC, Stephan W, Seidel M, Kuhn JMM, Haberer G, Tellier A. The de novo reference genome and transcriptome assemblies of the wild tomato species *Solanum chilense* highlights birth and death of NLR genes between tomato species. G3. 2019;9(12):3933–3941. 31604826 10.1534/g3.119.400529 PMC6893187

[49] Kahlon PS, Verin M, Hückelhoven R, Stam R. Quantitative resistance differences between and within natural populations of *Solanum chilense* against the oomycete pathogen *Phytophthora infestans*. Ecol Evol. 2021;11(12):7768–7778. 34188850 10.1002/ece3.7610 PMC8216925

[50] Soltis NE, Atwell S, Shi G, Fordyce R, Gwinner R, Gao D, Shafi A, Kliebenstein DJ. Interactions of tomato and *Botrytis cinerea* genetic diversity: Parsing the contributions of host differentiation, domestication, and pathogen variation. Plant Cell. 2019;31(2):502–519. 30647076 10.1105/tpc.18.00857 PMC6447006

[51] Boland GJ, Hall R. Index of plant hosts of *Sclerotinia sclerotiorum*. Can J Plant Pathol. 1994;16(2):93–108.

[52] Derbyshire MC, Denton-Giles M. The control of sclerotinia stem rot on oilseed rape (*Brassica napus*): Current practices and future opportunities. Plant Pathol. 2016;65(6):859–877.

[53] Mazumdar P. Sclerotinia stem rot in tomato: A review on biology, pathogenicity, disease management and future research priorities. J Plant Dis Prot. 2021;128:1403–1431.

[54] O’Sullivan CA, Belt K, Thatcher LF. Tackling control of a cosmopolitan phytopathogen: *Sclerotinia*. Front Plant Sci. 2021;12: Article 707509. 34490008 10.3389/fpls.2021.707509 PMC8417578

[55] Chen J, Ullah C, Giddings Vassão D, Reichelt M, Gershenzon J, Hammerbacher A. *Sclerotinia sclerotiorum* infection triggers changes in primary and secondary metabolism in *Arabidopsis thaliana*. Phytopathology. 2021;111(3):559–569. 32876531 10.1094/PHYTO-04-20-0146-R

[56] Sucher J, Mbengue M, Dresen A, Barascud M, Didelon M, Barbacci A, Raffaele S. Phylotranscriptomics of the Pentapetalae reveals frequent regulatory variation in plant local responses to the fungal pathogen *Sclerotinia sclerotiorum*. Plant Cell. 2020;32(6):1820–1844. 32265317 10.1105/tpc.19.00806 PMC7268813

[57] Uloth MB, You MP, Finnegan PM, Banga SS, Banga SK, Sandhu PS, Yi H, Salisbury PA, Barbetti MJ. New sources of resistance to *Sclerotinia sclerotiorum* for crucifer crops. Field Crop Res. 2013;154:40–52.

[58] Wei D, Mei J, Fu Y, Disi JO, Li J, Qian W. Quantitative trait loci analyses for resistance to *Sclerotinia sclerotiorum* and flowering time in *Brassica napus*. Mol Breed. 2014;34:1797–1804.

[59] Williams B, Kabbage M, Kim H-J, Britt R, Dickman MB. Tipping the balance: *Sclerotinia sclerotiorum* secreted oxalic acid suppresses host defenses by manipulating the host redox environment. PLoS Pathog. 2011;7(6): Article e1002107. 21738471 10.1371/journal.ppat.1002107 PMC3128121

[60] Wang Z, Ma L-Y, Cao J, Li Y-L, Ding L-N, Zhu K-M, Yang Y-H, Tan X-L. Recent advances in mechanisms of plant defense to *Sclerotinia sclerotiorum*. Front Plant Sci. 2019;10:1314. 31681392 10.3389/fpls.2019.01314 PMC6813280

[61] Badet T, Voisin D, Mbengue M, Barascud M, Sucher J, Sadon P, Balagué C, Roby D, Raffaele S. Parallel evolution of the POQR prolyl oligo peptidase gene conferring plant quantitative disease resistance. PLoS Genet. 2017;13(12): Article e1007143. 29272270 10.1371/journal.pgen.1007143 PMC5757927

[62] Li R, Rimmer R, Buchwaldt L, Sharpe AG, Séguin-Swartz G, Coutu C, Hegedus DD. Interaction of *Sclerotinia sclerotiorum* with a resistant *Brassica napus* cultivar: Expressed sequence tag analysis identifies genes associated with fungal pathogenesis. Fungal Genet Biol. 2004;41(8):735–753. 15219559 10.1016/j.fgb.2004.03.001

[63] Gehan MA, Fahlgren N, Abbasi A, Berry JC, Callen ST, Chavez L, Doust AN, Feldman MJ, Gilbert KB, Hodge JG, et al. PlantCV v2: Image analysis software for high-throughput plant phenotyping. PeerJ. 2017;5: Article e4088. 29209576 10.7717/peerj.4088 PMC5713628

[64] R Core Team, *R: A language and environment for statistical computing*. Vienna (Austria): R Foundation for Statistical Computing; 2022. https://www.R-project.org/.

[65] Wickham H, Averick M, Bryan J, Chang W, McGowan LD, François R, Grolemund G, Hayes A, Henry L, Hester J, et al. Welcome to the tidyverse. J Open Source Softw. 2019;4(43):1686.

[66] Wickham H. *ggplot2*. New York (NY): Springer New York; 2009. 10.1007/978-0-387-98141-3

[67] Kassambara A. ggpubr: “ggplot2” Based Publication Ready Plots. 2023. https://CRAN.R-project.org/package=ggpubr

[68] de Mendiburu F, Yaseen M. Agricolae: Statistical procedures for agricultural research; 2020. https://CRAN.R-project.org/package=agricolae

[69] Carroll RJ, Ruppert D. Transformation and weighting in regression New York: Chapman and Hall; 1988.

[70] McCullagh P, Nelder JA. Generalized linear models. 2nd ed. [Nachdr.]. London: Chapman & Hall; 1999.

[71] Nakagawa S, Schielzeth H. A general and simple method for obtaining *R*^2^ from generalized linear mixed-effects models. Methods Ecol Evol. 2013;4(2):133–142.

[72] Bretz F, Hothorn T, Westfall P. *Multiple comparisons using R*. 0 ed. Chapman and Hall/CRC; 2016.

[73] Hothorn T, Bretz F, Westfall P. Simultaneous inference in general parametric models. Biom J. 2008;50(3):346–363. 18481363 10.1002/bimj.200810425

[74] Caseys C, Shi G, Soltis N, Gwinner R, Corwin J, Atwell S, Kliebenstein DJ. Quantitative interactions: The disease outcome of *Botrytis cinerea* across the plant kingdom. G3. 2021;11(8):jkab175. 34003931 10.1093/g3journal/jkab175 PMC8496218

[75] Badet T, Léger O, Barascud M, Voisin D, Sadon P, Vincent R, Le Ru A, Balagué C, Roby D, Raffaele S. Expression polymorphism at the *ARPC 4* locus links the actin cytoskeleton with quantitative disease resistance to *Sclerotinia sclerotiorum* in *Arabidopsis thaliana*. New Phytol. 2019;222(1):480–496. 30393937 10.1111/nph.15580

[76] Foolad R, Zhang P, Khan AA, Niño-Liu D, Lin Y. Identification of QTLs for early blight (*Alternaria solani*) resistance in tomato using backcross populations of a *Lycopersicon esculentum* × *L. hirsutum* cross. Theor Appl Genet. 2002;104(6-7):945–958. 12582599 10.1007/s00122-002-0870-z

[77] Kabas A, Fidan H, Kucukaydin H, Atan HN. Screening of wild tomato species and interspecific hybrids for resistance/tolerance to tomato brown rugose fruit virus (ToBRFV). Chil J Agric Res. 2022;82(1):189–196.

[78] Stam R, Scheikl D, Tellier A. The wild tomato species *Solanum chilense* shows variation in pathogen resistance between geographically distinct populations. PeerJ. 2017;5: Article e2910. 28133579 10.7717/peerj.2910 PMC5248578

[79] Zhang LP, Lin GY, Niño-Liu D, Foolad MR. Mapping QTLs conferring early blight (*Alternaria solani*) resistance in a *Lycopersicon esculentum* × *L. hirsutum* cross by selective genotyping. Mol Breed. 2003;12(1):3–19.

[80] Méline V, Caldwell DL, Kim B, Khangura RS, Baireddy S, Yang C, Sparks EE, Dilkes B, Delp EJ, Iyer-Pascuzzi AS. Image-based assessment of plant disease progression identifies new genetic loci for resistance to *Ralstonia solanacearum* in tomato. Plant J. 2023;113(5):887–903. 36628472 10.1111/tpj.16101

[81] Ding L-N, Li T, Guo X-J, Li M, Liu X-Y, Cao J, Tan X-L. Sclerotinia stem rot resistance in rapeseed: Recent progress and future prospects. J Agric Food Chem. 2021;69(10):2965–2978. 33667087 10.1021/acs.jafc.0c07351

[82] Peralta IE, Spooner DM, Knapp S. Taxonomy of wild tomatoes and their relatives (*Solanum* sect. *Lycopersicoides*, sect *Juglandifolia*, sect *Lycopersicon*; Solanaceae). Am Soc Plant Taxonomists. 2008;84:186.

[83] Lambrechts L, Fellous S, Koella JC. Coevolutionary interactions between host and parasite genotypes. Trends Parasitol. 2006;22(1):12–16. 16310412 10.1016/j.pt.2005.11.008

[84] Pedras MSC, Hossain S, Snitynsky RB. Detoxification of cruciferous phytoalexins in *Botrytis cinerea*: Spontaneous dimerization of a camalexin metabolite. Phytochemistry. 2011;72(2-3):199–206. 21176925 10.1016/j.phytochem.2010.11.018

[85] Chauhan S, Katoch S, Sharma SK, Sharma PN, Rana JC, Singh K, Singh M. Screening and identification of resistant sources against *Sclerotinia sclerotiorum* causing white mold disease in common bean. Crop Sci. 2020;60(4):1986–1996.

[86] Yanar Y, Miller SA. Resistance of pepper cultivars and accessions of *capsicum* spp. to *Sclerotinia sclerotiorum*. Plant Dis. 2003;87(3):303–307. 30812765 10.1094/PDIS.2003.87.3.303

[87] Kahlon PS, Seta SM, Zander G, Scheikl D, Hückelhoven R, Joosten MHAJ, Stam R. Population studies of the wild tomato species *Solanum chilense* reveal geographically structured major gene-mediated pathogen resistance. Proc Biol Sci. 2020;287(1941):20202723. 33352079 10.1098/rspb.2020.2723 PMC7779489

[88] Stam R, Silva-Arias GA, Tellier A. Subsets of NLR genes show differential signatures of adaptation during colonization of new habitats. New Phytol. 2019;224(1):367–379. 31230368 10.1111/nph.16017

[89] Rick CM, Potential genetic resources in tomato species: Clues from observations in native habitats. In: Srb AM, editor. *Genes, enzymes and populations*. Boston (MA): Springer US; 1973. p. 255–269. 10.1007/978-1-4684-2880-3_17 4792359

[90] Fordyce RF, Soltis NE, Caseys C, Gwinner R, Corwin JA, Atwell S, Copeland D, Feusier J, Subedy A, Eshbaugh R, et al. Digital imaging combined with genome-wide association mapping links loci to plant-pathogen interaction traits. Plant Physiol. 2018;178(3):1406–1422. 30266748 10.1104/pp.18.00851 PMC6236616

[91] Soltis NE, Caseys C, Zhang W, Corwin JA, Atwell S, Kliebenstein DJ. Pathogen genetic control of transcriptome variation in the *Arabidopsis thaliana*–*Botrytis cinerea* pathosystem. Genetics. 2020;215(1):253–266. 32165442 10.1534/genetics.120.303070 PMC7198280

[92] Kahlon PS, Förner A, Muser M, Oubounyt M, Gigl M, Hammerl R, Baumbach J, Hückelhoven R, Dawid C, Stam R. Laminarin-triggered defence responses are geographically dependent in natural populations of *Solanum chilense*. J Exp Bot. 2023;74(10):erad087. 10.1093/jxb/erad087 PMC10199122 36880316

[93] Altmann M, Altmann S, Rodriguez PA, Weller B, Elorduy Vergara L, Palme J, Marín-de La Rosa N, Sauer M, Wenig M, Villaécija-Aguilar JA, et al. Extensive signal integration by the phytohormone protein network. Nature. 2020;583(7815):271–276. 32612234 10.1038/s41586-020-2460-0

[94] Kahlon PS, Stam R. Polymorphisms in plants to restrict losses to pathogens: From gene family expansions to complex network evolution. Curr Opin Plant Biol. 2021;62: Article 102040. 33882435 10.1016/j.pbi.2021.102040

[95] Mutka AM, Fentress SJ, Sher JW, Berry JC, Pretz C, Nusinow DA, Bart R. Quantitative, image-based phenotyping methods provide insight into spatial and temporal dimensions of plant disease. Plant Physiol. 2016;172(2):650–660. 27443602 10.1104/pp.16.00984 PMC5047107

[96] Stam R, McDonald BA. When resistance gene pyramids are not durable—The role of pathogen diversity: R-gene pyramid durability and pathogen diversity. Mol Plant Pathol. 2018;19(3):521–524. 29446883 10.1111/mpp.12636 PMC6637985

