# Supplementary material for: High-Resolution Disease Phenotyping Reveals Distinct Resistance Mechanisms of Tomato Crop Wild Relatives against Sclerotinia sclerotiorum
Source: Plant Phenomics. 2024 Aug 5;6:0214. doi: 10.34133/plantphenomics.0214 (PMC11298253; doi:10.34133/plantphenomics.0214)
Supplement: Supplementary 1 — Figs. S1 to S9 Tables S1 to S5 [file plantphenomics.0214.f1.pdf]

SCIENCE PARTNER JOURNALS

Supplementary Materials for:

High-resolution disease phenotyping reveals distinct resistance mechanisms of wild tomato crop wild relatives against *Sclerotinia sclerotiorum*

Short title: Wild tomatoes harbour unique QDR mechanisms

In: Plant Phenomics

Authors

Severin Einspanier[1], Christopher Tominello-Ramirez[1], Mario Hasler[2], Adelin Barbacci[3], Sylvain Raffaele[3], Remco Stam[1*]

[1] Department of Phytopathology and Crop Protection, Institute of Phytopathology, Faculty of Agricultural and Nutritional Sciences, Christian Albrechts University, Kiel, Germany

[2] Lehrfach Variationsstatistik, Faculty of Agricultural and Nutritional Sciences, Christian-Albrechts-University of Kiel, Hermann-Rodewald-Straße 9, 24118 Kiel, Germany

[3] Laboratoire des Interactions Plantes Microorganismes Environnement (LIPME), INRAE, CNRS, Castanet Tolosan Cedex, France.

* Address correspondence to: Remco.Stam@phytomed.uni-kiel.de

Supplementary Material

Tables

Suppl. Table 1 Estimate Means lag-phase duration per species.

Suppl. Table 2: Estimate Means LDT

Suppl. Table 3: Grand Mean contrasts lag

Suppl. Table 4: Grand Mean contrasts LDT

Suppl. Table 5: *Solanum* accessions used in this study

Figures

Suppl. Figure 1: Exemplary schematic of the "navautron" phenotyping platform.

Suppl. Figure 2: Lag-phase duration shows different levels of variation depending on the host species.

Suppl. Figure 3: LDT shows different levels of variation depending on the host species.

Suppl. Figure 4: Per-accession infection frequency estimates. Values derived from a glm on three independent repetitions.

Suppl. Figure 5: Correlation analysis between Infection frequency estimates and LDT.

Suppl. Figure 6: Pooled correlation analysis of all accessions.

Suppl. Figure 7: Bright light microscopy images of *S. sclerotiorum* infections on two *S. pennellii* accessions with different lag phase durations.

Suppl. Figure 8: Residual plot of lag-phase duration values.

Suppl. Figure 9: Residual plot of LDT values.

Tables

Suppl. Table 1: Estimate Means lag-phase duration per species.

Species	Lag estimate [h]	std.error [h]
S. habrochaites	59.74	3.10
S. lycopersicoides	43.32	2.71
S. pennellii	59.87	2.71
S. pimpinellifolium	36.22	0.89

Suppl. Table 2: Estimate Means LDT

Species	LDT-estimate [h]	std.error [h]
S. habrochaites	36.85	0.02
S. lycopersicoides	41.13	0.02
S. pennellii	11.80	0.02
S. pimpinellifolium	11.77	0.02

Suppl. Table 3: Grand Mean contrasts lag

Species	Accession	estimate (h)	std.error	statistic	adj.p.value	signif
S. habrochaites	LA1721	-13.9805	3.2319	-0.0721	0.0006	***
S. habrochaites	LA2167	-12.0752	3.2768	-0.0614	0.0078	**
S. habrochaites	LA1559	-8.2475	2.3949	-0.0574	0.0191	*
S. habrochaites	LA1731	-5.0888	3.3181	-0.0256	0.9840	ns
S. habrochaites	LA2128	-0.1378	5.7960	-0.0004	1.0000	ns
S. habrochaites	LA2409	-0.0536	2.5831	-0.0003	1.0000	ns
S. habrochaites	LA1753	15.8582	5.5225	0.0479	0.1275	ns
S. habrochaites	LA2864	23.7252	6.5906	0.0600	0.0107	*
S. pennellii	LA1809	-17.0236	1.6687	-0.1700	0.0000	***
S. pennellii	LA2657	-6.3068	1.3466	-0.0781	0.0001	***
S. pennellii	LA1941	-5.4652	1.7853	-0.0510	0.0713	.
S. pennellii	LA2963	-3.1692	3.2787	-0.0161	1.0000	ns
S. pennellii	LA2719	-0.3337	1.5238	-0.0036	1.0000	ns
S. pennellii	LA1282	1.5361	1.3216	0.0194	0.9998	ns
S. pennellii	LA0716	6.5780	1.8289	0.0599	0.0108	*
S. pennellii	LA1656	10.7843	1.8850	0.0954	0.0000	***
S. pennellii	LA1303	13.4000	1.9487	0.1146	0.0000	***
S. lycopersicoides	LA2772	-7.6031	1.5262	-0.0830	0.0000	***
S. lycopersicoides	LA2776	-5.5762	1.2054	-0.0771	0.0001	***
S. lycopersicoides	LA4130	-2.4261	1.5880	-0.0255	0.9847	ns
S. lycopersicoides	LA2777	1.4128	1.5828	0.0149	1.0000	ns
S. lycopersicoides	LA1964	4.4474	1.4488	0.0512	0.0693	.
S. lycopersicoides	LA4123	4.6261	1.4813	0.0520	0.0584	.
S. lycopersicoides	LA2951	5.1190	1.9937	0.0428	0.2865	ns
S. pimpinellifolium	LA1593	-7.8207	0.8582	-0.1519	0.0000	***
S. pimpinellifolium	LA2853	-6.1720	1.5283	-0.0673	0.0018	**
S. pimpinellifolium	LA1374	-6.0821	1.1340	-0.0894	0.0000	***
S. pimpinellifolium	LA1332	-5.4925	1.1039	-0.0829	0.0000	***
S. pimpinellifolium	LA1261	-3.5010	1.6940	-0.0344	0.7161	ns
S. pimpinellifolium	LA1659	-0.5469	0.8171	-0.0112	1.0000	ns
S. pimpinellifolium	LA4713	4.6669	4.1684	0.0187	0.9999	ns
S. pimpinellifolium	LA2347	5.5052	1.0240	0.0896	0.0000	***
S. pimpinellifolium	LA1348	9.0943	1.1659	0.1300	0.0000	***
S. pimpinellifolium	LA2983	10.3488	1.5771	0.1094	0.0000	***

Suppl. Table 4: Grand Mean contrasts LDT

Species	Accession	Estimate	Std.Error	Statistic	Adj.p.value	signif.
S. habrochaites	LA2128	-0.3450	0.0984	-3.5052	0.0154	*
S. habrochaites	LA2167	-0.1418	0.0797	-1.7798	0.9157	ns
S. habrochaites	LA2409	-0.1036	0.0609	-1.7024	0.9466	ns
S. habrochaites	LA1731	-0.0322	0.0661	-0.4866	1.0000	ns
S. habrochaites	LA1753	0.0102	0.0890	0.1143	1.0000	ns
S. habrochaites	LA1559	0.0349	0.0619	0.5633	1.0000	ns
S. habrochaites	LA1721	0.1810	0.0837	2.1633	0.6332	ns
S. habrochaites	LA2864	0.3965	0.1654	2.3970	0.4207	ns
S. lycopersicoides	LA2777	-0.5631	0.0319	-17.6649	0.0000	***
S. lycopersicoides	LA4130	-0.3304	0.0469	-7.0464	0.0000	***
S. lycopersicoides	LA2776	-0.1336	0.0311	-4.2920	0.0006	***
S. lycopersicoides	LA4123	-0.1313	0.0450	-2.9176	0.1119	ns
S. lycopersicoides	LA2772	0.1880	0.0298	6.3131	0.0000	***
S. lycopersicoides	LA1964	0.4522	0.0423	10.6881	0.0000	***
S. lycopersicoides	LA2951	0.5182	0.0463	11.2031	0.0000	***
S. pennellii	LA1303	-0.2561	0.0324	-7.9098	0.0000	***
S. pennellii	LA2657	-0.1336	0.0229	-5.8337	0.0000	***
S. pennellii	LA1656	-0.1202	0.0396	-3.0352	0.0779	.
S. pennellii	LA1282	-0.0897	0.0230	-3.8936	0.0033	**
S. pennellii	LA1809	-0.0774	0.0267	-2.8946	0.1202	ns
S. pennellii	LA2719	-0.0714	0.0271	-2.6405	0.2405	ns
S. pennellii	LA0716	-0.0397	0.0288	-1.3792	0.9967	ns
S. pennellii	LA1941	0.2596	0.0501	5.1787	0.0000	***
S. pennellii	LA2963	0.5285	0.0445	11.8656	0.0000	***
S. pimpinellifolium	LA2983	-0.2359	0.0571	-4.1295	0.0013	**
S. pimpinellifolium	LA4713	-0.1162	0.0878	-1.3232	0.9983	ns
S. pimpinellifolium	LA1374	-0.0457	0.0302	-1.5143	0.9871	ns
S. pimpinellifolium	LA2853	-0.0209	0.0555	-0.3770	1.0000	ns
S. pimpinellifolium	LA2347	-0.0174	0.0356	-0.4882	1.0000	ns
S. pimpinellifolium	LA1659	-0.0172	0.0294	-0.5839	1.0000	ns
S. pimpinellifolium	LA1593	0.0622	0.0307	2.0227	0.7566	ns
S. pimpinellifolium	LA1332	0.0895	0.0302	2.9637	0.0972	.
S. pimpinellifolium	LA1348	0.1126	0.0319	3.5288	0.0142	*
S. pimpinellifolium	LA1261	0.1890	0.0741	2.5516	0.2996	ns

Suppl. Table 5 *Solanum* accessions used in this study

Accession	Solanum species
**LA0716**	*pennellii*
**LA1261**	*pimpinellifolium*
**LA1282**	*pennellii*
**LA1303**	*pennellii*
**LA1332**	*pimpinellifolium*
**LA1348**	*pimpinellifolium*
**LA1374**	*pimpinellifolium*
**LA1559**	*habrochaites*
**LA1593**	*pimpinellifolium*
**LA1656**	*pennellii*
**LA1659**	*pimpinellifolium*
**LA1721**	*habrochaites*
**LA1731**	*habrochaites*
**LA1753**	*habrochaites*
**LA1809**	*pennellii*
**LA1941**	*pennellii*
**LA1964**	*lycopersicoides*
**LA2128**	*habrochaites*
**LA2167**	*habrochaites*
**LA2347**	*pimpinellifolium*
**LA2409**	*habrochaites*
**LA2657**	*pennellii*
**LA2719**	*pennellii*
**LA2772**	*lycopersicoides*
**LA2776**	*lycopersicoides*
**LA2777**	*lycopersicoides*
**LA2853**	*pimpinellifolium*
**LA2864**	*habrochaites*
**LA2951**	*lycopersicoides*
**LA2963**	*pennellii*
**LA2983**	*pimpinellifolium*
**LA4123**	*lycopersicoides*
**LA4130**	*lycopersicoides*
**LA4713**	*pimpinellifolium*
**C32**	*lycopersicum*

Figures

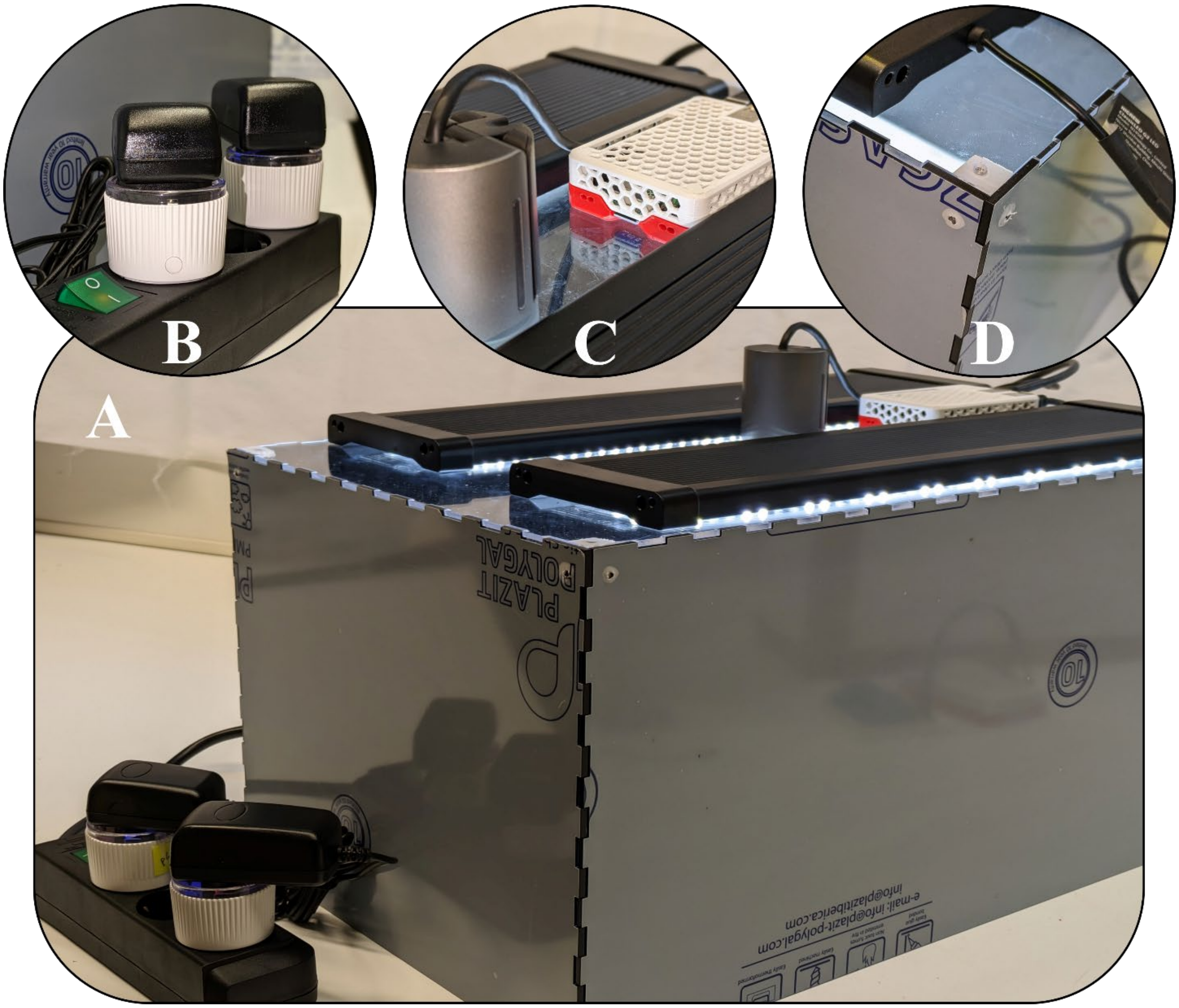

Suppl. figure 1: **Exemplary schematic of the "navautron" phenotyping platform.** The phenotyping system consists of a Poly(methyl methacrylate) box with custom fittings (D), LED growth lights, WiFi-smart plugs (B), and a 4k camera (C). A Raspberry Pi micro-computer controls the lights and the image acquisition (C). The raw images are stored on a separate file server. The PMMA-box can be customized to fit individual needs.

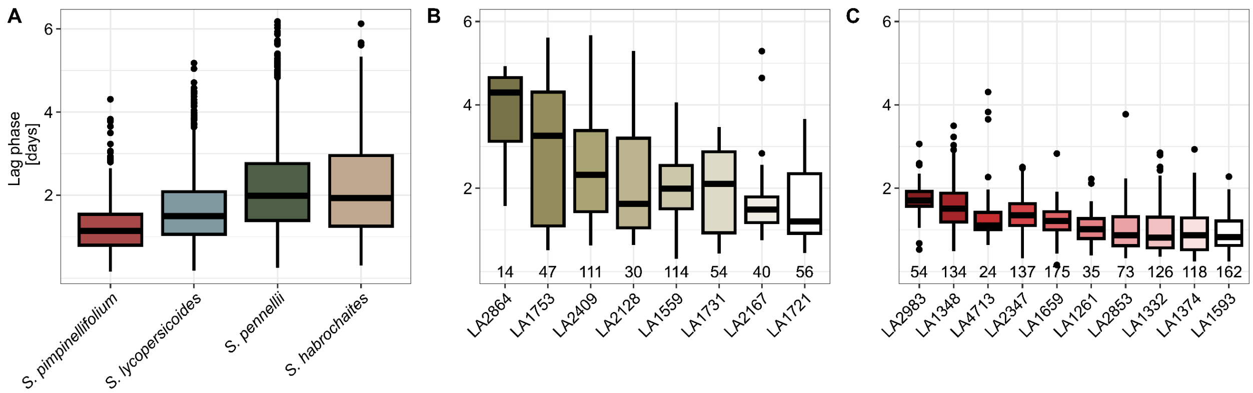

Suppl. Figure 2: **Lag-phase duration shows different levels of variation depending on the host species.** A) The lag phase duration (in days after infection) of *S. sclerotiorum* infection on *S. pennellii, S. lycopersicoides, S. pimpinellifolium* and *S. habrochaites* accessions. (B)Lag-phase duration of *S. habrochaites* accessions and C) *S. pimpinellifolium* accessions. The number on the x-axis indicates the count of individual leaves tested.

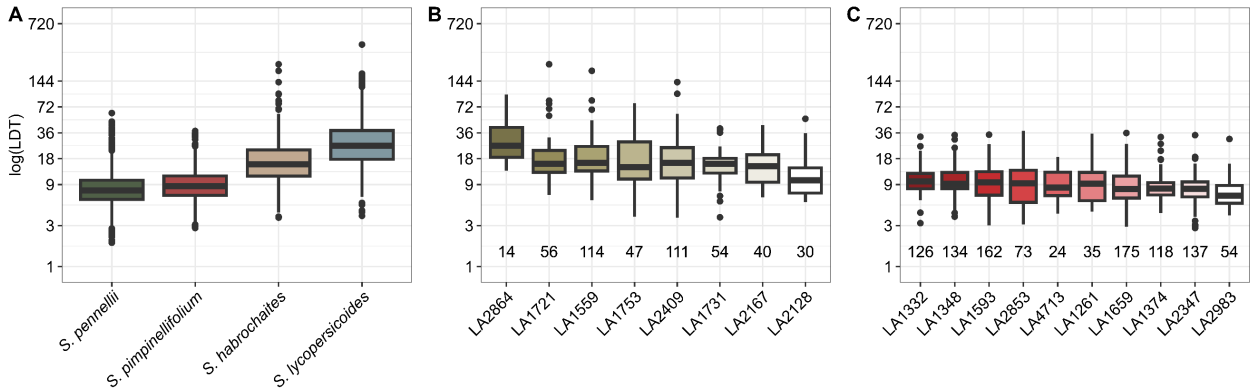

Suppl. Figure 3: **LDT shows different levels of variation depending on the host species.** A) The LDT (in hours) of *S. sclerotiorum* infection on *S. pennellii, S. lycopersicoides, S. pimpinellifolium* and *S. habrochaites* accessions. (B) LDT of *S. habrochaites* accessions and C) *S. pimpinellifolium* accessions. The number on the x-axis indicates the count of individual leaves tested.

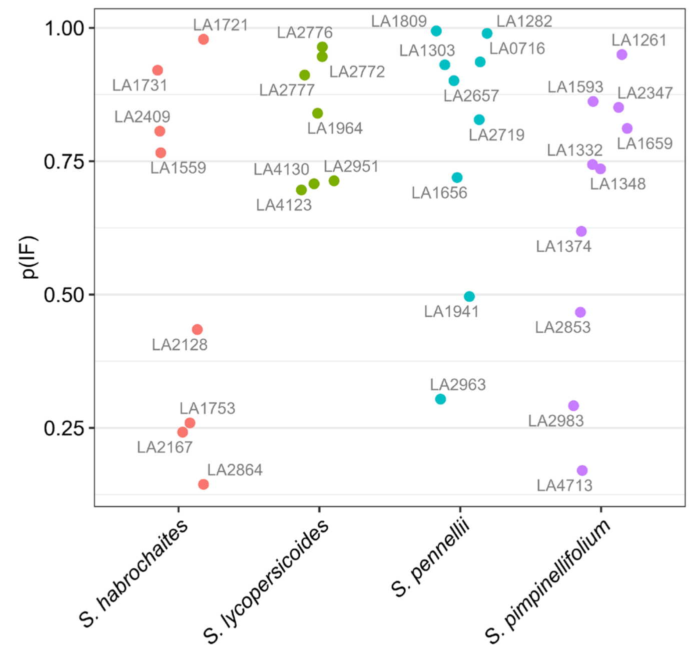

Suppl. Figure 4: **Per-accession infection frequency estimates. Values derived from a glm on three independent repetitions.**

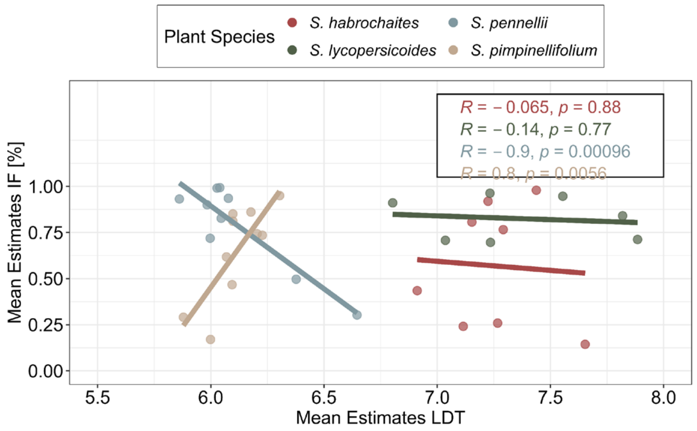

Suppl. Figure 5: **Correlation analysis between Infection frequency estimates and LDT.**

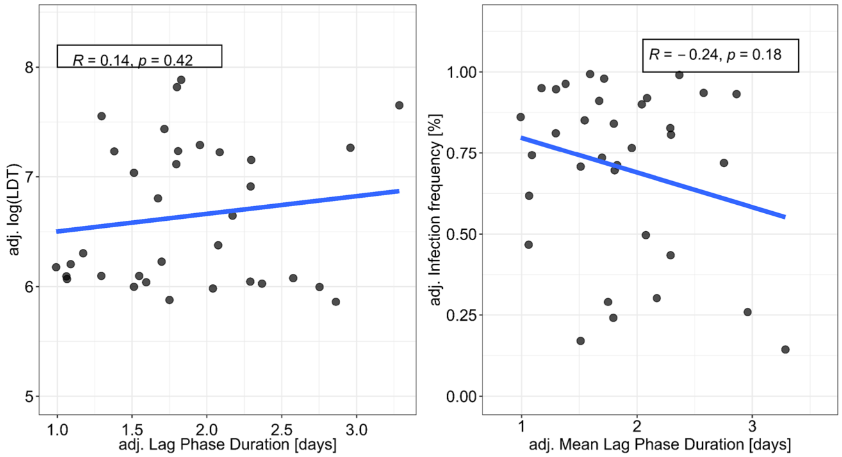

Suppl. Figure 6: **Pooled correlation analysis of all accessions.**

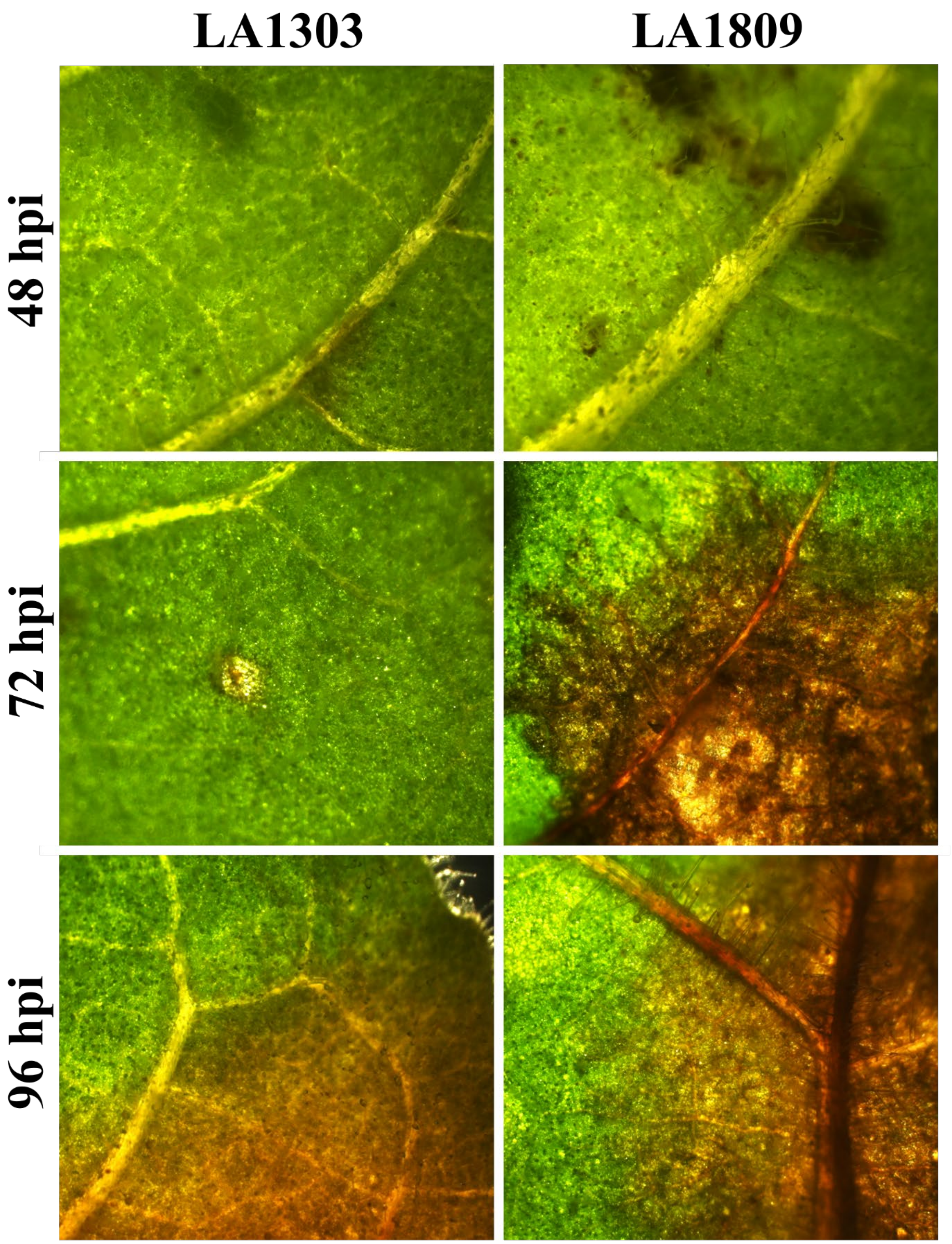

Suppl. Figure 7: **Bright light microscopy images of *S. sclerotiorum* infections on two *S. pennellii* accessions with different lag phase durations.**

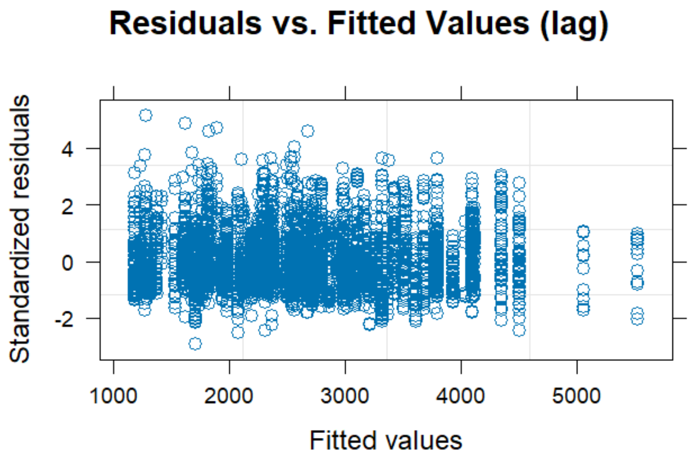

Suppl. Figure 8: **Residual plot of lag-phase duration values.**

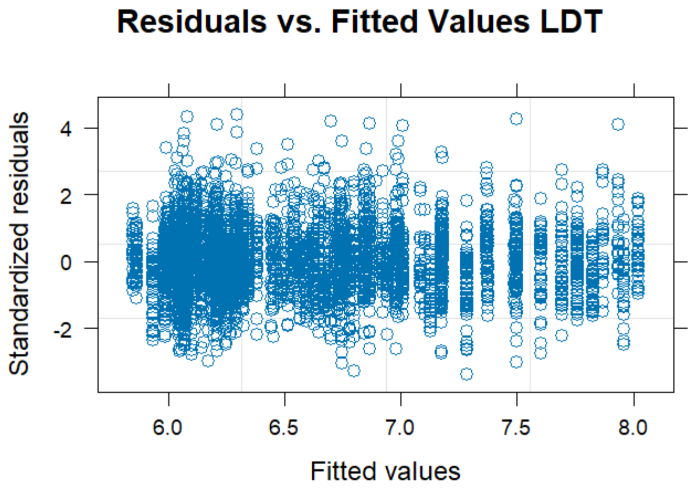

Suppl. Figure 9: **Residual plot of LDT values.**
